# Cytochrome b5 reductase orchestrates IL-1β production in macrophages through FAD

**DOI:** 10.1038/s41419-025-08073-2

**Published:** 2025-10-21

**Authors:** Jian Fu, Zhihua Liu, Hangchao Zhang, Xinmei Zhang, Shijie Liu, Xuehua Mei, Xiu Zeng, Wenkai Ren

**Affiliations:** 1 State Key Laboratory of Swine and Poultry Breeding Industry, Guangdong Laboratory of Lingnan Modern Agriculture, College of Animal Science, South China Agricultural University, Guangzhou, China; 2 National Center of Technology Innovation for Pigs, Chongqing, China

**Keywords:** Monocytes and macrophages, Cell signalling

## Abstract

The cytochrome-b5 reductases (CYB5Rs) regulate cellular redox balance and contribute to the pathogenesis of inflammatory diseases. However, the roles of CYB5R5 in macrophages remain poorly understood and require further elucidation. In this study, we revealed that CYB5R5 orchestrates macrophage inflammation by inhibiting interleukin (IL)-1β production from M1 macrophages. Mechanistically, CYB5R5 enhances flavin adenine dinucleotide (FAD)-lysine demethylase1 (LSD1) signaling to regulate the histone demethylation of complement component 1, q subcomponent (C1q)-coding genes, thereby lowering NLRP3 inflammasome assembly. We also found that myeloid depletion of *Cyb5r5* in mice exacerbates inflammatory responses in LPS-induced sepsis. This study reveals that CYB5R5 attenuates M1 macrophage polarization via metabolic and epigenetic reprogramming mechanism, thus providing potential therapeutic targets for macrophage-mediated inflammatory disorders.

## Introduction

Flavoprotein cytochrome b5 reductases (CYB5Rs) function as canonical electron shuttles in redox homeostasis, orchestrating NADH-dependent electron transfer via CYB5-mediated relay to terminal metabolic substrates [1]. Mammalian CYB5Rs comprise five isoforms (CYB5R1-5). Among these, CYB5R5 is unequivocally the least characterized, exhibiting the lowest sequence identity to the well-studied CYB5R3 isoform [1]. Critically, emerging evidence links CYB5R5 dysregulation to various inflammatory diseases [2, 3], suggesting a potential regulatory role in systemic immune responses beyond its canonical metabolic functions. However, the specific function of CYB5R5 within immune cell compartments, particularly in macrophages—key orchestrators of inflammation—remains completely unexplored. Given its identity as a flavoprotein utilizing FAD [1], CYB5R5 is poised to influence not only redox metabolism but potentially also FAD-dependent epigenetic processes, positioning it as a compelling candidate for regulating macrophage inflammatory responses.

Macrophages are critical in innate immunity and characterized by their remarkable phenotypic plasticity, enabling them to perform a broad range of functions in response to different stimuli [4]. These functions can be classified into M1/M2 macrophage paradigm [5]. Under lipopolysaccharides (LPS) or interferon-gamma (IFN-γ)-induced inflammatory conditions, peripheral blood monocytes are chemotactically recruited to inflammatory foci, undergoing polarization into pro-inflammatory M1 macrophages. These M1 macrophages produce cytokines like interleukin (IL)-1β, contributing to the inflammatory responses [6]. Conversely, macrophages can also polarize into a M2 phenotype, which is essential for tissue regeneration and reparative processes [7]. Dysregulation of the transition between these phenotypes, such as prolonged activation of M1 macrophages, is implicated in various macrophage-related diseases such as sepsis [8–10]. Therefore, understanding the regulatory mechanisms behind macrophage polarization is of great interest, with a focus on cellular signaling pathways, metabolism, and epigenetic factors. Cellular metabolic remodeling is pivotal in shaping specialized phenotypic characteristics of macrophages [11]. CYB5Rs critically regulate cellular redox equilibrium and catalyze diverse redox-related biochemical processes that influence metabolism, including the desaturation of saturated fatty acids and cholesterol biosynthesis [1]. This suggests a correlation between CYB5Rs and M1 macrophage polarization. However, a critical knowledge gap persists in elucidating the role of CYB5Rs, particularly CYB5R5, in modulating macrophage phenotypes.

In this study, we demonstrate that CYB5R5 inhibits M1 macrophage IL-1β production. Mechanistically, CYB5R5 enhances the signaling of flavin adenine dinucleotide (FAD)-dependent lysine-specific demethylase 1 (LSD1), which in turn regulates the histone demethylation of complement component 1, q subcomponent (C1q)-coding genes to reduce NLRP3 inflammasome assembly and IL-1β production. Our findings indicate that CYB5R5 has the potential to mitigate M1 macrophage responses through metabolic and epigenetic reprogramming, thereby offering new therapeutic targets for macrophage-mediated inflammatory disorders.

## Results

### CYB5R5 is expressed in M1 macrophages

**Fig. 1:**
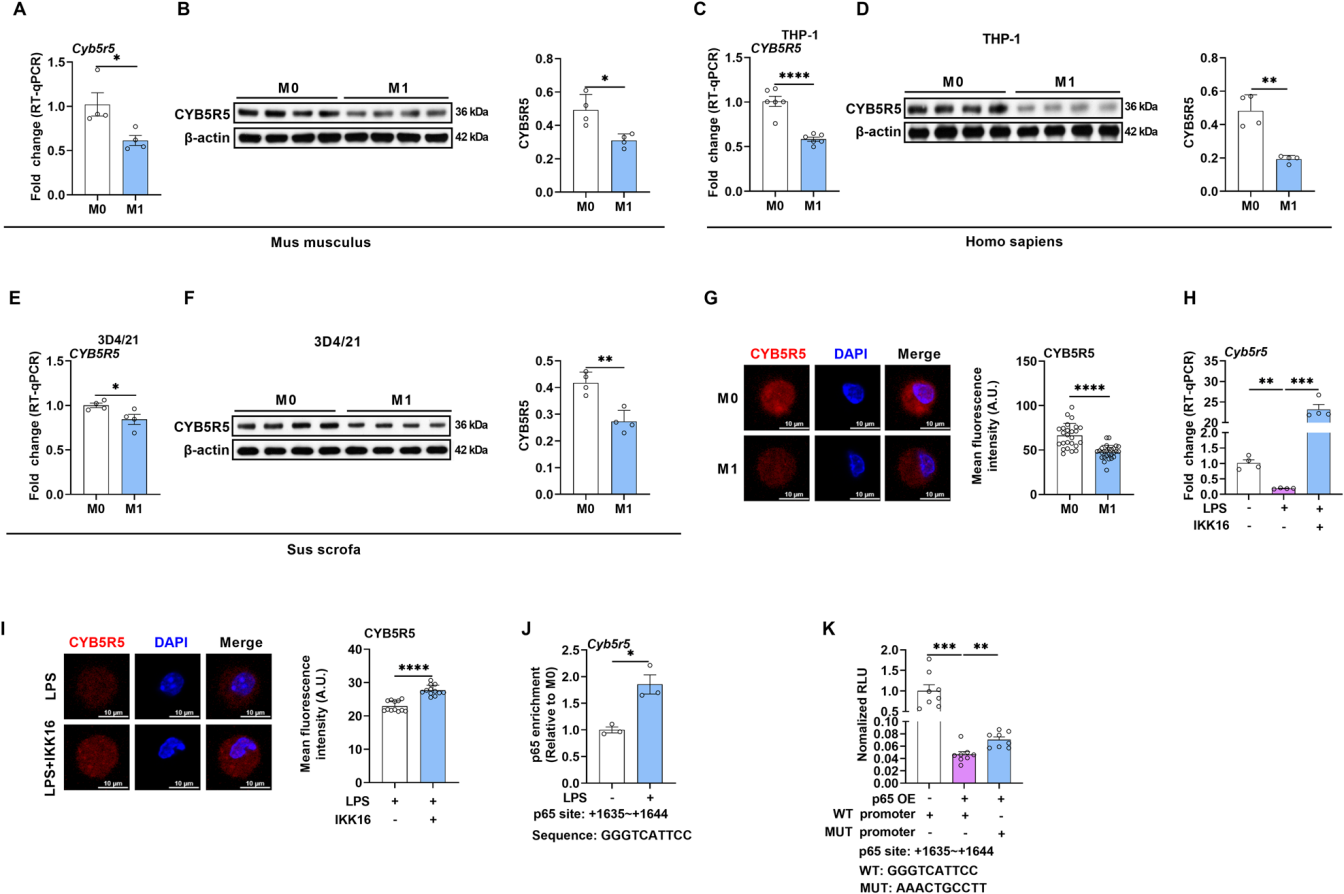
LPS inhibits CYB5R5 expression by NF-κB signaling pathway. **A** Relative mRNA expression of *Cyb5r5* in M0 and M1 of peripheral macrophages (PEMs) (n = 4). **B** Relative protein abundance of CYB5R5 in M0 and M1 of PEMs (n = 4). **C** Relative mRNA expression of *CYB5R5* in M0 and M1 of THP-1 (n = 6). **D** Relative protein abundance of CYB5R5 in M0 and M1 of THP-1 (n = 4). **E** Relative mRNA expression of *CYB5R5* in M0 and M1 of 3D4/21 (n = 4). **F** Relative protein abundance of CYB5R5 in M0 and M1 of 3D4/21 (n = 4). **G** Immunofluorescence (IF) analysis of CYB5R5 in PEMs (n = 6 samples and observed 2–3 cells per sample). **H** Relative mRNA expression of *Cyb5r5* in PEMs treated with or without LPS or LPS plus IKK16 (n = 4). **I** IF analysis of CYB5R5 in PEMs treated with LPS or LPS plus IKK16 (n = 6 samples and observed 2–3 cells per sample). **J** ChIP-qPCR analysis of the p65 occupancy at the promoter of *Cyb5r5* in PEMs (n = 3). **K** Dual-Luciferase analysis of p65 binding to wild type *Cyb5r5* or mutant *Cyb5r5* promoter (n = 8). In this and subsequent figures, M0 refers to macrophages cultured without any treatment, and M1 denotes macrophages stimulated with LPS and IFN-γ, unless otherwise specified. Data were analyzed with unpaired t-test (**A**–**G**, **I**, **J**) or one-way ANOVA (**H**, **K**) and represented with mean ± SD other than RT-qPCR results. The n value represents replicates unless indicated. *p < 0.05, **p < 0.01, ***p < 0.001, ****p < 0.0001.

Unlike its well-characterized homolog (CYB5R3), CYB5R5 remains poorly studied. With referencing to UniProt, CYB5R proteins shared the same structural motifs, while CYB5R5 shared the least sequence identity with CYB5R3 (Fig. S1A). Although CYB5R5 is associated with various inflammatory diseases [12], the expression profile of CYB5R5 in macrophages remains uncharacterized. To substantiate this, we induced M1 polarization based on previous study [13]. The mRNA expression and protein abundance of CYB5R5 in M1 of peritoneal macrophages (PEMs) decreased at 12 hours (Fig. 1A, B). Moreover, previous RNA-seq data [14] showed that *Cyb5r5* expression decreased in M1 macrophages (Fig. S1B). Given that more than 80% sequence identity of CYB5R5 among various species (Fig. S1C), we investigated CYB5R5 expression in other species. Similarly, *Cyb5r5* expression and CYB5R5 abundance decreased in M1 macrophages of human macrophage cells (THP-1) (Fig. 1C, D) and porcine alveolar macrophage cells (3D4/21) (Fig. 1E, F). Notably, *Cyb5r5* expression was consistently inhibited in various infectious models by analyzing GEO Datasets (Fig. S1D–F). Moreover, transcriptional profiling revealed an inverse regulatory relationship between CYB5R5 and canonical M1 polarization markers, including IL-1β, CD86, CCL2, CCL3, CXCL1, CXCL10, STAT1 and IL-6 (Fig. S1G, H).

The CYB5R5 was predominantly localized in the cytoplasm of macrophages (Fig. 1G). Subsequent investigations were directed towards delineating the molecular cascade responsible for the LPS/IFN-γ-induced transcriptional repression of *Cyb5r5*. Notably, LPS stimulation alone potently suppressed *Cyb5r5* expression in PEMs, and the inhibition of LPS-induced NF-κB signaling pathway activation through IKK-16 [15] completely restored *Cyb5r5* expression (Fig. 1H, I). ChIP-qPCR analysis also confirmed LPS-induced enrichment of p65 at the *Cyb5r5* promoter (Fig. 1J). Additionally, the transcriptional regulation of *Cyb5r5* by NF-κB signaling was validated using dual-luciferase reporter assays (Fig. 1K). Collectively, these findings indicate that LPS inhibits *Cyb5r5* expression in M1 macrophages.

We also examined CYB5R5 expression in M2 macrophages. Interestingly, IL-4 stimulation (associated with M2a macrophage polarization) significantly decreased *Cyb5r5* expression and CYB5R5 abundance in PEMs at 12 hours (Fig. S1I, J). Further investigation is required to elucidate the mechanism underlying these observations.

### CYB5R5 regulates M1 macrophage polarization

**Fig. 2:**
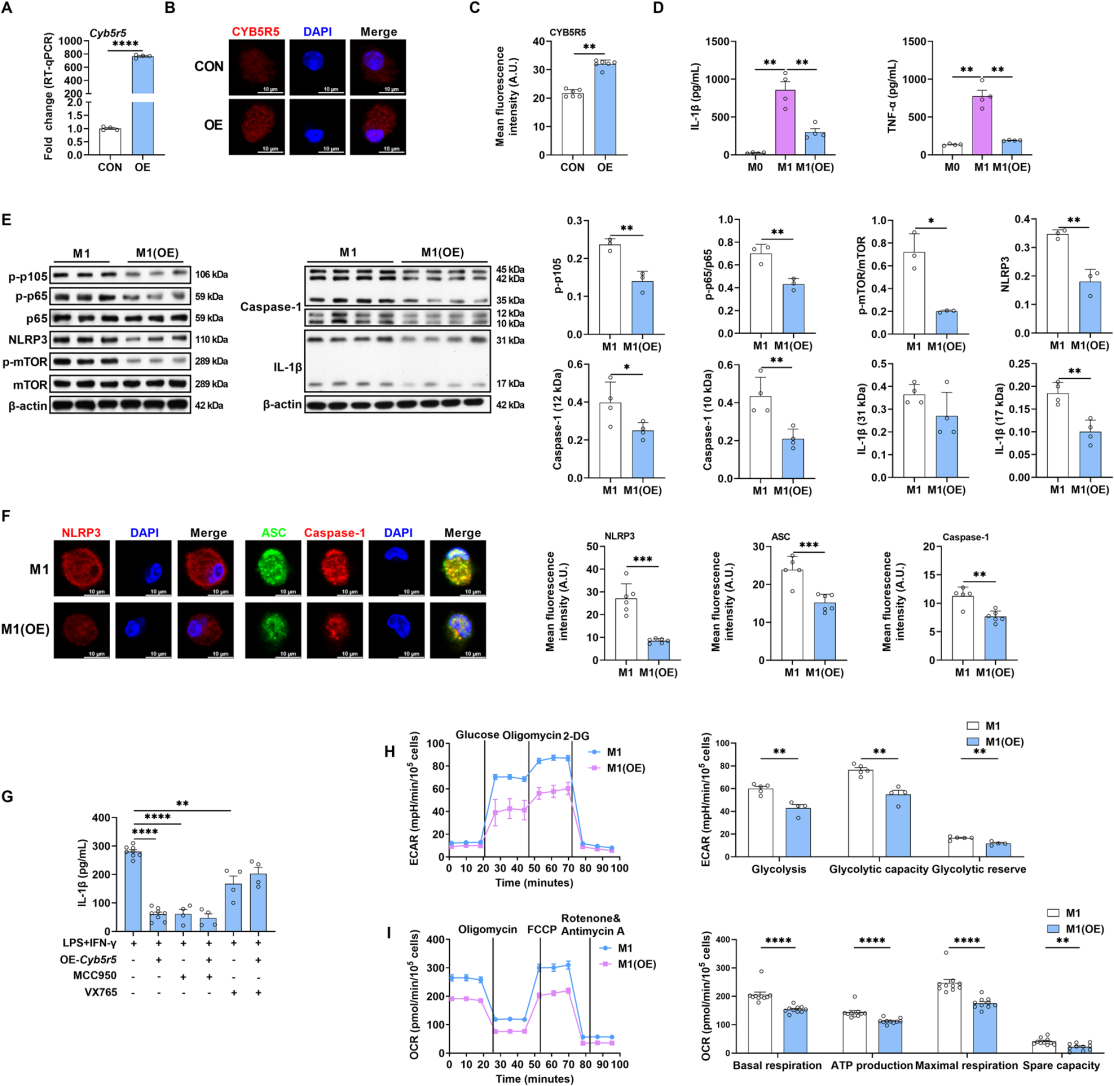
CYB5R5 suppresses M1 macrophage polarization. **A** Relative mRNA expression of *Cyb5r5* in PEMs with CYB5R5 overexpression (n = 4). **B**, **C** IF analysis of CYB5R5 in PEMs with CYB5R5 overexpression (n = 6). **D** The secretion of IL-1β and TNF-α from PEMs treated with CYB5R5 overexpression (n = 4). **E** The protein abundance of p-p105, p-p65, p65, NLRP3, p-mTOR, mTOR, Caspase-1, and IL-1β in M1 with CYB5R5 overexpression (n = 3–4). **F** IF analysis of NLRP3 (red, left), Caspase-1 (red, right), and ASC (green, right) in M1 with CYB5R5 overexpression (n = 5–6). **G** The secretion of IL-1β from M1 treated with or without CYB5R5 overexpression in the presence of MCC950 or VX765 (n = 4–8). **H** The ECAR including glycolysis, glycolytic capacity and glycolytic reserve in M1 with CYB5R5 overexpression (n = 4–5). **I** The OCR including the basal respiration, ATP production, maximal respiration and spare capacity in M1 with CYB5R5 overexpression (n = 10). Data were analyzed with unpaired t-test (**A**, **C**, **E**, **F**, **H**, **I**) or one-way ANOVA (**D**, **G**) and represented with mean ± SD other than RT-qPCR results. *p < 0.05, **p < 0.01, ***p < 0.001, ****p < 0.0001.

The observed downregulation of CYB5R5 in M1 macrophages prompted investigation into its potential regulatory role in polarization dynamics. We then overexpressed CYB5R5 in macrophages (Fig. 2A–C). CYB5R5 overexpression significantly suppressed the production of IL-1β (a marker of M1 macrophages) as well as TNF-α in M1 macrophages polarized from PEMs (Fig. 2D). Similar reduction in IL-1β and TNF-α production after CYB5R5 overexpression was also observed in M1 macrophages polarized from ANA.1 cells (mouse macrophage cell line) and bone marrow-derived macrophages (BMDMs) (Fig. S2A, B). Correspondently, silencing *Cyb5r5* with small interfering RNA promoted IL-1β and TNF-α production in M1 macrophages polarized from PEMs (Fig. S2C, D).

M1 macrophage polarization involves coordinated activation of NF-κB signaling, inflammasome, and mTOR signaling [13]. CYB5R5 overexpression inhibited the activation of NF-κB signaling, inflammasome, and mTOR signaling, as evidenced by the reduction in the ratio of p-p65/p65 and p-mTOR/mTOR, as well as the abundance of p-p105, NLRP3, cleaved Caspase-1, and IL-1β (Fig. 2E), and the assembly of the inflammasome in M1 macrophages (Fig. 2F). To further validate the role of inflammasome in CYB5R5-mediated IL-1β production, we analyzed IL-1β production in CYB5R5-overexpressing M1 macrophages in the presence of inflammasome inhibitors (MCC950 and VX765). Notably, CYB5R5 overexpression failed to reduce IL-1β production in the presence of these inflammasome inhibitors (Fig. 2G). Mitochondrial functions, including dynamics and respiratory activity, are also implicated in IL-1β production in M1 macrophages [16]. CYB5R5 overexpression in M1 macrophages affected mitochondrial biomass (Fig. S2E), abundance of proteins associated with mitochondrial fission/fusion (Fig. S2F), extracellular acidification rates (ECAR, an indicator of glycolysis) (Fig. 2H), and oxygen consumption rates (OCR, an indicator of oxidative phosphorylation) (Fig. 2I). To further confirm the involvement of mitochondrial function in CYB5R5-mediated IL-1β production, we analyzed IL-1β production in CYB5R5-overexpressing M1 macrophages in the presence of mitochondrial function inhibitors (Antimycin A, Oligomycin, and DMM). Notably, CYB5R5 overexpression still reduced IL-1β production in the presence of these mitochondrial function inhibitors (Fig. S2G). Collectively, CYB5R5 is negatively correlated with M1 macrophage IL-1β production.

We also investigated whether CYB5R5 influences M2 macrophage polarization. Overexpression of CYB5R5 resulted in reduced abundance of IL-10, p-STAT6/STAT6, ARG-1, and CD206 in M2a macrophages (Fig. S2H). However, the role of CYB5R5 in M2 macrophage polarization warrants further investigation.

### CYB5R5 mediates M1 macrophage IL-1β production through C1q

**Fig. 3:**
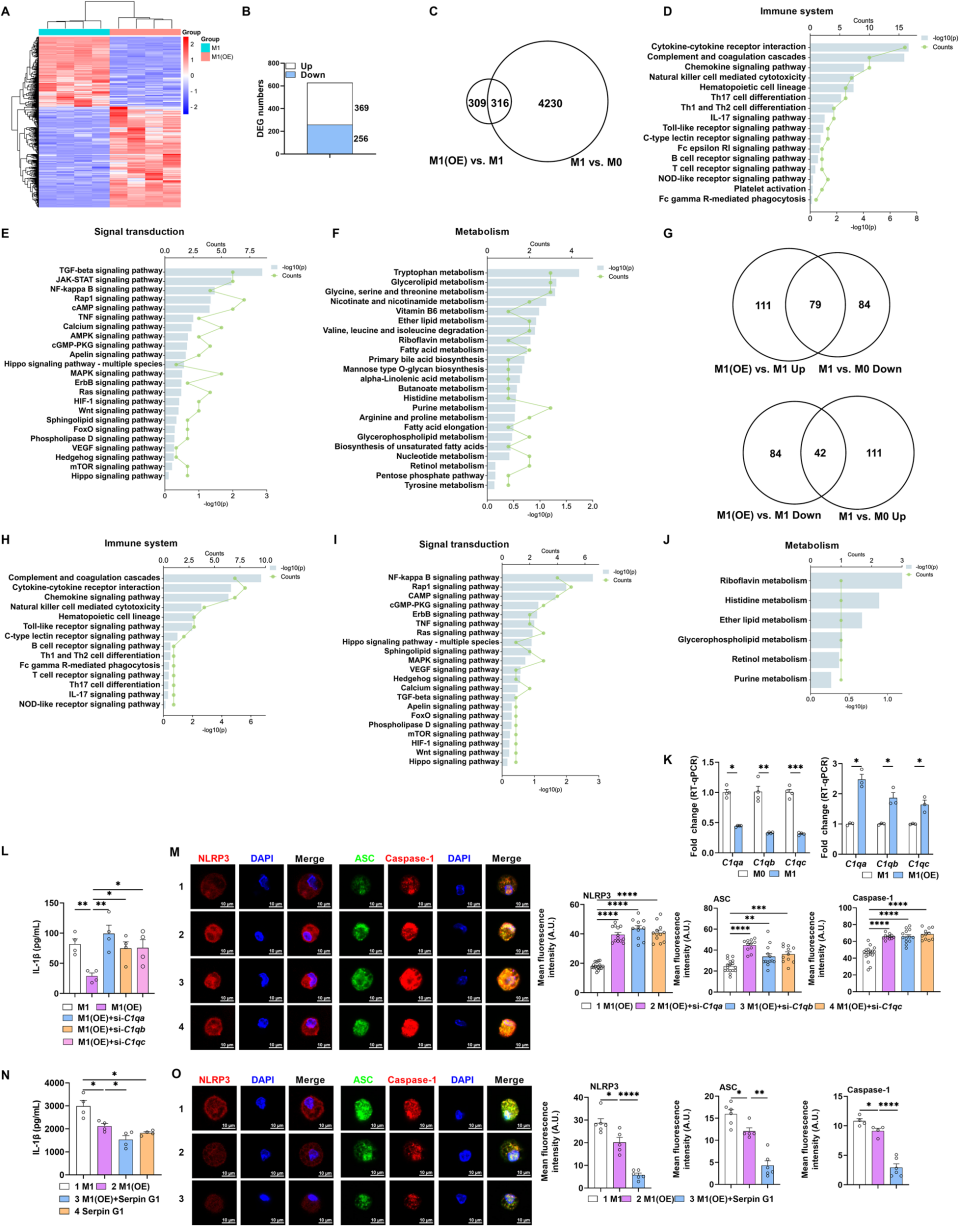
CYB5R5 suppresses M1 macrophage polarization via C1q. **A** Heatmap analysis of differential expression genes (DEGs) in M1 macrophages with CYB5R5 overexpression (n = 4). **B** Histogram of DEGs (fold change > 2; *p* < 0.05) in M1 macrophages with CYB5R5 overexpression. **C** Venn of common DEGs (fold change >2; *p* < 0.05) between M1(OE) vs. M1 and M1 vs. M0 groups. **D**–**F** Histogram of DEGs in **C** enriched in immune system, signal transduction and metabolism. **G** Venn of common DEGs with inverse change (fold change >2; p < 0.05) between M1(OE) vs. M1 and M1 vs. M0 groups. **H**–**J** Histogram of DEGs in **G** enriched in immune system, signal transduction and metabolism. **K** Relative mRNA expression of C1q-coding genes in PEMs between M1 vs. M0 and M1(OE) vs. M1 groups (n = 3–4). **L** The secretion of IL-1β from M1 treated with CYB5R5 overexpression or CYB5R5 overexpression plus si-*C1qa*, si-*C1qb* or si-*C1qc* (n = 4). **M** IF analysis of NLRP3 (red, left), Caspase-1 (red, right), and ASC (green, right) in PEMs treated as **L** (n = 6 samples and observed 2–3 cells per sample). **N** The secretion of IL-1β from M1 treated with CYB5R5 overexpression or CYB5R5 overexpression plus Serpin G1 or Serpin G1 (n = 4). **O** IF analysis of NLRP3 (red, left), Caspase-1 (red, right), and ASC (green, right) in PEMs treated as **N** (n = 4–6). Data were analyzed with unpaired t-test (**K**) or one-way ANOVA (**L**–**O**) and represented with mean ± SD other than RT-qPCR results. *p < 0.05, **p < 0.01, ***p < 0.001, ****p < 0.0001.

To uncover the underlying mechanism of CYB5R5-mediated IL-1β production, comparative transcriptomic profiling via RNA-seq was conducted between M1 macrophages (M1) and M1 macrophages with CYB5R5 overexpression (M1(OE)). CYB5R5 overexpression remodeled macrophage transcriptomic architecture (Figs. 3A, B and S3A, B). Consistent with the aforementioned results (Fig. 2), the differentially expressed genes (DEGs) after CYB5R5 overexpression were enriched in signaling pathways (e.g., NF-κB, inflammasome, and mTOR) (Fig. S3C–E). By cross-referencing these DEGs with genes identified during M1 macrophage polarization in our prior work [14], we subsequently identified 316 common DEGs (Fig. 3C), which were enriched in immune system, metabolism and signaling pathways (e.g., NF-κB, inflammasome, and mTOR) (Fig. 3D–F). Collectively, CYB5R5 overexpression significantly reshapes M1 macrophage transcriptomic profile, especially enriched in signaling pathways, such as NF-κB, inflammasome, and mTOR.

To identify the critical DEGs that influence CYB5R5-mediated IL-1β production in M1 macrophages, candidate DEGs were selected based on the following criteria: 1) increased in M1(OE) versus M1 while decreased in M1 versus M0; 2) decreased in M1(OE) versus M1 while increased in M1 versus M0. Using these criteria, a total of 121 DEGs were identified, which were primarily enriched in immune system, metabolism and signaling pathways such as NF-κB, inflammasome, and mTOR (Figs. 3G–J and S3F). Among these DEGs, five genes including *Ccr5*, *Cxcr3*, *Cxcl9*, *Lck*, and *C1qb*, were selected for further study through string interactions (Fig. S3G). All selected genes except *Lck* showed consistent expression changes in both RNA-Seq and RT-qPCR assays (Figs. 3K and S3H). Besides, among the up-regulated genes (*Ccr5*, *Cxcr3*, and *C1qb*), only *C1qb*, encoding the C1q protein, shows a negative correlation with M1 macrophage polarization [17–19]. Conversely, down-regulated gene *Cxcl9* exhibits a positive correlation with M1 macrophage polarization but acts through interacting with CXCR3 [20, 21]. Moreover, C1q consists of 6 identical subunits, each of which contains 3 polypeptide chains encoded by *C1qa*, *C1qb*, and *C1qc* [22]. Thus, *C1qa*, *C1qb*, and *C1qc* were selected for further study.

We hypothesized that CYB5R5 upregulates the expression of *C1qa*, *C1qb*, and *C1qc* to reduce IL-1β production. To test this hypothesis, we silenced *C1qa*, *C1qb*, or *C1qc* in the presence of CYB5R5 overexpression and observed that IL-1β production was fully restored after silencing *C1qa*, *C1qb*, or *C1qc* (Figs. 3L and S3I). Silencing *C1qa*, *C1qb*, or *C1qc* also rescued the effects of CYB5R5 overexpression on the inflammasome (Fig. 3M). Serpin G1 inhibits C1q degradation [23]. Thus, we activated C1q in the presence of CYB5R5 overexpression using Serpin G1 and found that IL-1β production further decreased after C1q activation (Figs. 3N and S3J). C1q activation also further inhibited the activation of inflammasome (Fig. 3O). Collectively, CYB5R5 upregulates the expression of C1q-coding genes to reduce IL-1β production in M1 macrophages.

### CYB5R5 mediates M1 macrophage IL-1β production through FAD-LSD1-C1q signaling

**Fig. 4:**
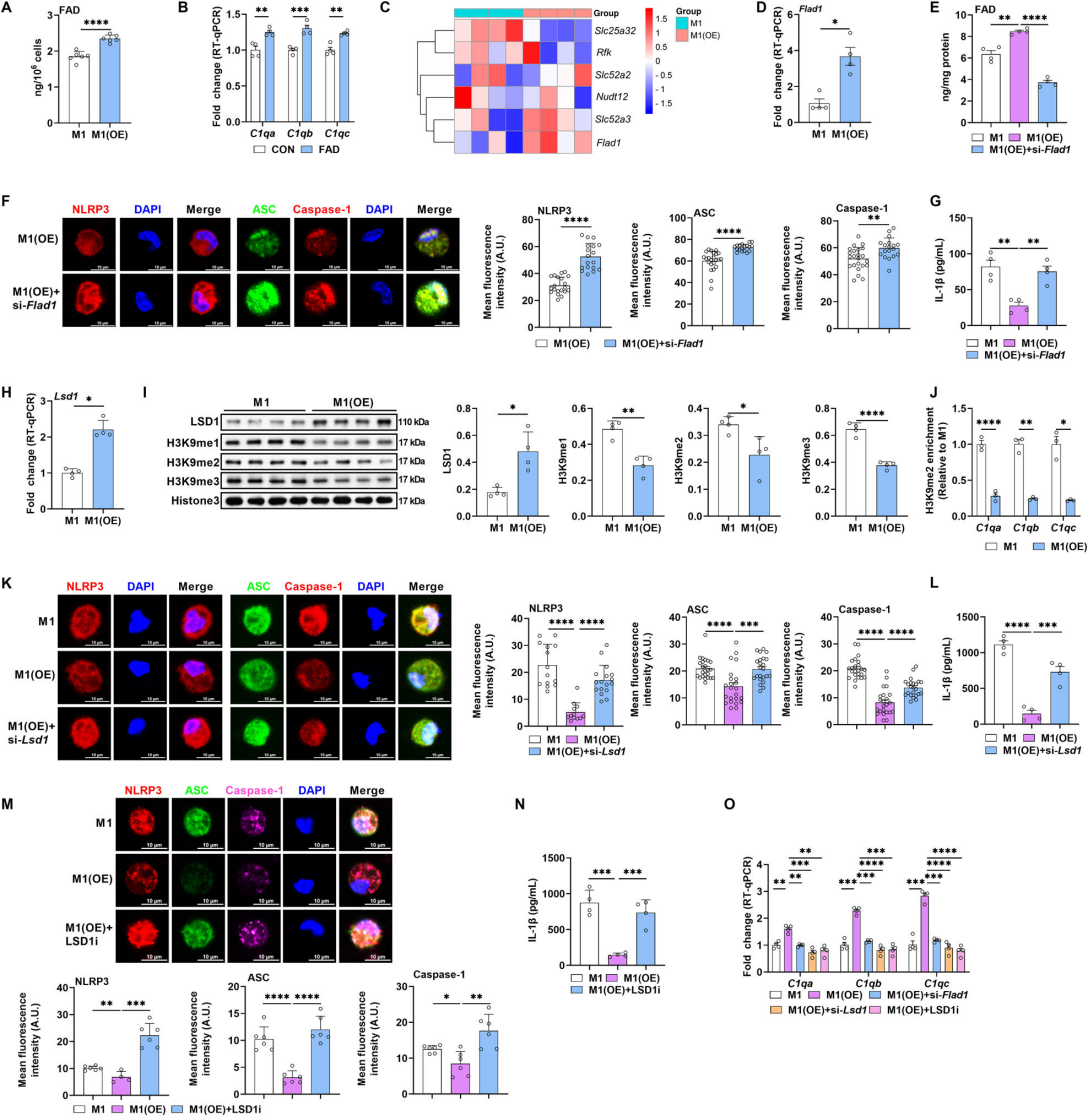
CYB5R5 suppresses M1 macrophage polarization via FAD-LSD1-C1q axis. **A** The levels of FAD in M1 macrophages with CYB5R5 overexpression (n = 6). **B** Relative mRNA expression of C1q-coding genes in PEMs treated with FAD (n = 4). **C** Heatmap analysis of genes related to FAD metabolism and transportation in M1 macrophages with *Cyb5r5* overexpression (n = 4). **D** Relative mRNA expression of *Flad1* in M1 macrophages with CYB5R5 overexpression (n = 4). **E** The levels of FAD in M1 macrophages treated with CYB5R5 overexpression or CYB5R5 overexpression plus si-*Flad1* (n = 4). **F** IF analysis of NLRP3 (red, left), Caspase-1 (red, right), and ASC (green, right) in PEMs treated as **E** (n = 6 samples and observed 2–3 cells per sample). **G** The secretion of IL-1β from PEMs treated as **E** (n = 4). **H** Relative mRNA expression of *Lsd1* in M1 macrophages with CYB5R5 overexpression (n = 4). **I** The protein abundance of LSD1, H3K9me1, H3K9me2 and H3K9me3 in M1 macrophages with CYB5R5 overexpression (n = 4). **J** CHIP-qPCR analysis of H3K9me2 enrichment at *C1qa, C1qb*, and *C1qc* (n = 3). **K** IF analysis of NLRP3 (red, left), Caspase-1 (red, right), and ASC (green, right) in M1 macrophages treated with CYB5R5 overexpression or CYB5R5 overexpression plus si-*Lsd1* (n = 6 samples and observed 2–3 cells per sample). **L** The secretion of IL-1β from PEMs treated as **K** (n = 4). **M** IF analysis of NLRP3 (red), ASC (green), and Caspase-1 (purple) in M1 macrophages treated with CYB5R5 overexpression or CYB5R5 overexpression plus LSD1i (n = 4-6). **N** The secretion of IL-1β from PEMs treated as **M** (n = 4). **O** Relative mRNA expression of *C1qa, C1qb, and C1qc* in M1 macrophages treated with CYB5R5 overexpression or CYB5R5 overexpression plus si-*Flad1*, si-*Lsd1 or* LSD1i (n = 4). Data were analyzed with unpaired t-test (**A**, **B**, **D**, **F**, **H**–**J**) or one-way ANOVA (**E**, **G**, **K**–**O**) and represented with mean ± SD other than RT-qPCR results. *p < 0.05, **p < 0.01, ***p < 0.001, ****p < 0.0001.

The 121 DEGs identified in Fig. 3G were enriched in riboflavin metabolism, and active riboflavin (flavin adenine dinucleotide, FAD) serves as an essential cofactor for both CYB5R5 and LSD1 [1, 24]. FAD activates LSD1, which canonically acts on H3K9me1/2/3 to orchestrate transcriptional programs [25, 26]. CYB5R5 overexpression increased FAD level in M1 macrophages (Fig. 4A), and FAD elevated the expression of C1q-coding genes in M1 macrophages (Fig. 4B). To elucidate how CYB5R5 influences FAD level in macrophages, we analyzed genes related to riboflavin metabolism from RNA-seq data in Fig. 3 and found that *Flad1* was upregulated in M1 macrophages following CYB5R5 overexpression, which was confirmed by RT-qPCR analysis (Figs. 4C, D and S4A). Notably, silencing *Flad1* affected the FAD level (Figs. 4E and S4B). *Flad1* silencing counteracted the impacts of CYB5R5 overexpression on the inflammasome and IL-1β production (Fig. 4F, G). Therefore, we postulated that CYB5R5 inhibits IL-1β production in M1 macrophages through FAD/LSD1-C1q signaling. Notably, CYB5R5 overexpression increased the mRNA expression and protein abundance of LSD1 and decreased the protein abundance of H3K9me1, H3K9me2, and H3K9me3 (Fig. 4H, I). H3K9me2 is a classical marker of transcriptional repression. Notably, CYB5R5 overexpression decreased H3K9me2 enrichment on C1q-coding genes (Fig. 4J). LSD1 silencing or inhibition rescued the effects of CYB5R5 overexpression on the inflammasome and IL-1β production (Fig. 4K–N). Furthermore, silencing *Flad1* or LSD1 inhibition blocked the effects of CYB5R5 overexpression on C1q-coding genes (Fig. 4O). Collectively, CYB5R5 limits IL-1β production in M1 macrophages through the activation of FAD-LSD1-C1q signaling pathway.

### Myeloid depletion of *Cyb5r5* promotes M1 macrophage IL-1β production

**Fig. 5:**
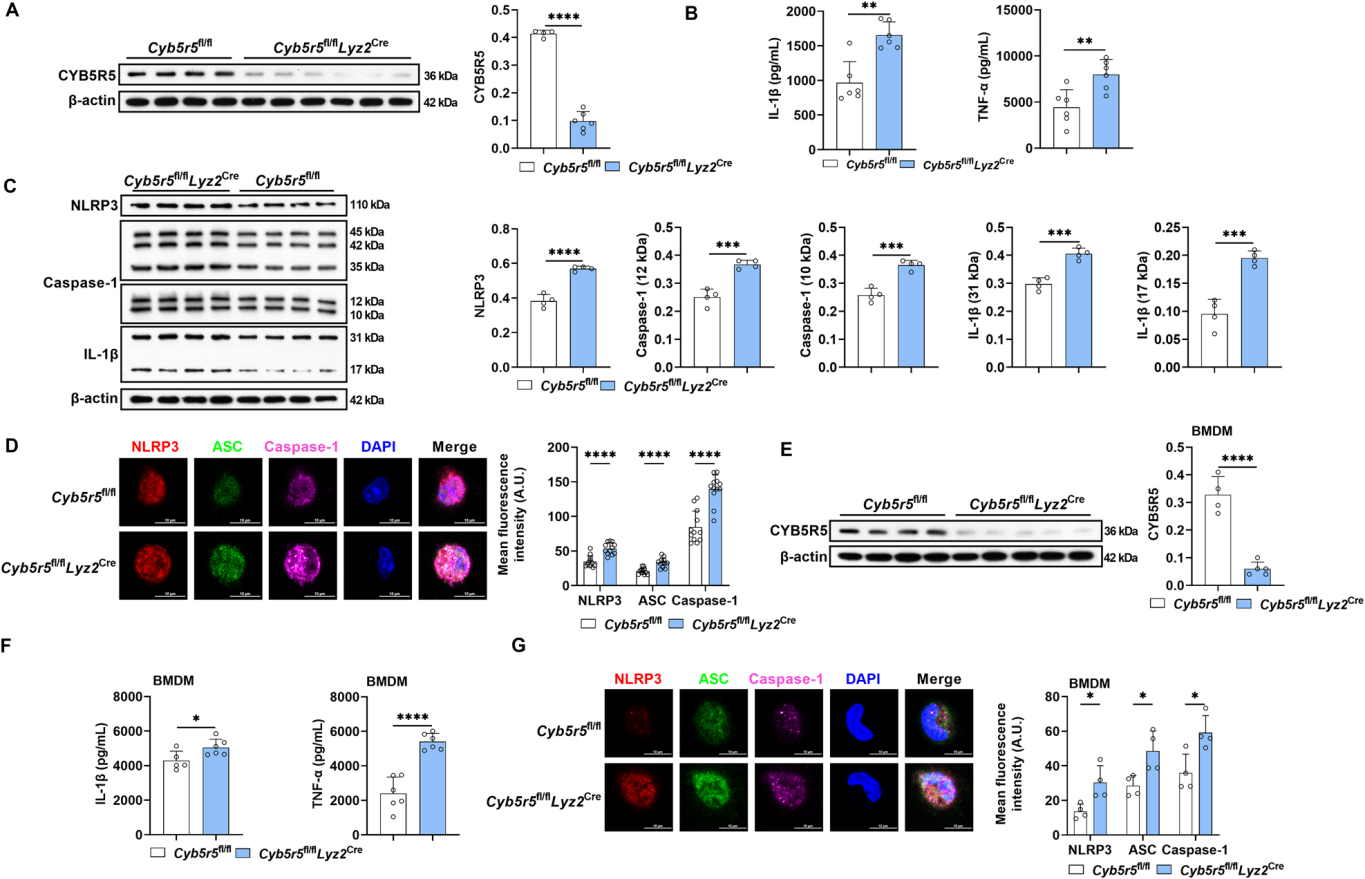
Myeloid depletion of *Cyb5r5* promotes M1 macrophage polarization. **A** The protein abundance of CYB5R5 in macrophages from *Cyb5r5*^fl/fl^ and *Cyb5r5*^fl/fl^ *Lyz2*^Cre^ mice (n = 4-6). **B** The secretion of IL-1β and TNF-α from M1 macrophages from *Cyb5r5*^fl/fl^ and *Cyb5r5*^fl/fl^ *Lyz2*^Cre^ mice (n = 6). **C** The protein abundance of NLRP3, Caspase-1, and IL-1β from M1 macrophages from *Cyb5r5*^fl/fl^ and *Cyb5r5*^fl/fl^ *Lyz2*^Cre^ mice (n = 4). **D** IF analysis of NLRP3 (red), ASC (green), and Caspase-1 (purple) from M1 macrophages from *Cyb5r5*^fl/fl^ and *Cyb5r5*^fl/fl^ *Lyz2*^Cre^ mice (n = 6 samples and observed 2–3 cells per sample). **E** The protein abundance of CYB5R5 in BMDMs from *Cyb5r5*^fl/fl^ and *Cyb5r5*^fl/fl^ *Lyz2*^Cre^ mice (n = 4–5). **F** The secretion of IL-1β and TNF-α from M1 BMDMs from *Cyb5r5*^fl/fl^ and *Cyb5r5*^fl/fl^ *Lyz2*^Cre^ mice (n = 5–6). **G** IF analysis of NLRP3 (red), ASC (green), and Caspase-1 (purple) from M1 BMDMs from *Cyb5r5*^fl/fl^ and *Cyb5r5*^fl/fl^ *Lyz2*^Cre^ mice (n = 4). Data were analyzed with unpaired t-test (**A**–**G**) and represented with mean ± SD. *p < 0.05, **p < 0.01, ***p < 0.001, ****p < 0.0001.

To further elucidate the regulatory role of CYB5R5 in macrophages, we crossed *Cyb5r5*^fl/fl^ mice (WT) with *Lyz2*^cre^ mice to produce mice with myeloid-specific *Cyb5r5* depletion (CKO) (Fig. S5A–C). The deletion of CYB5R5 in macrophages was confirmed (Figs. 5A and S5D). M1 macrophages from CKO PEMs exhibited higher levels of IL-1β and TNF-α (Fig. 5B), as well as increased abundance of NLRP3 inflammasome component (Fig. 5C), compared to WT M1 macrophages. The deletion of CYB5R5 also promoted NLRP3 inflammasome assembly in M1 macrophages (Fig. 5D). The deletion of CYB5R5 in BMDMs was also confirmed (Fig. 5E). Higher levels of IL-1β and TNF-α (Fig. 5F) and enhanced NLRP3 inflammasome assembly (Fig. 5G) were observed in M1 macrophages from CKO BMDMs. Collectively, myeloid depletion of *Cyb5r5* promotes M1 macrophage polarization.

### Myeloid depletion of *Cyb5r5* promotes M1 macrophage IL-1β production through FAD-LSD1-C1q signaling

**Fig. 6:**
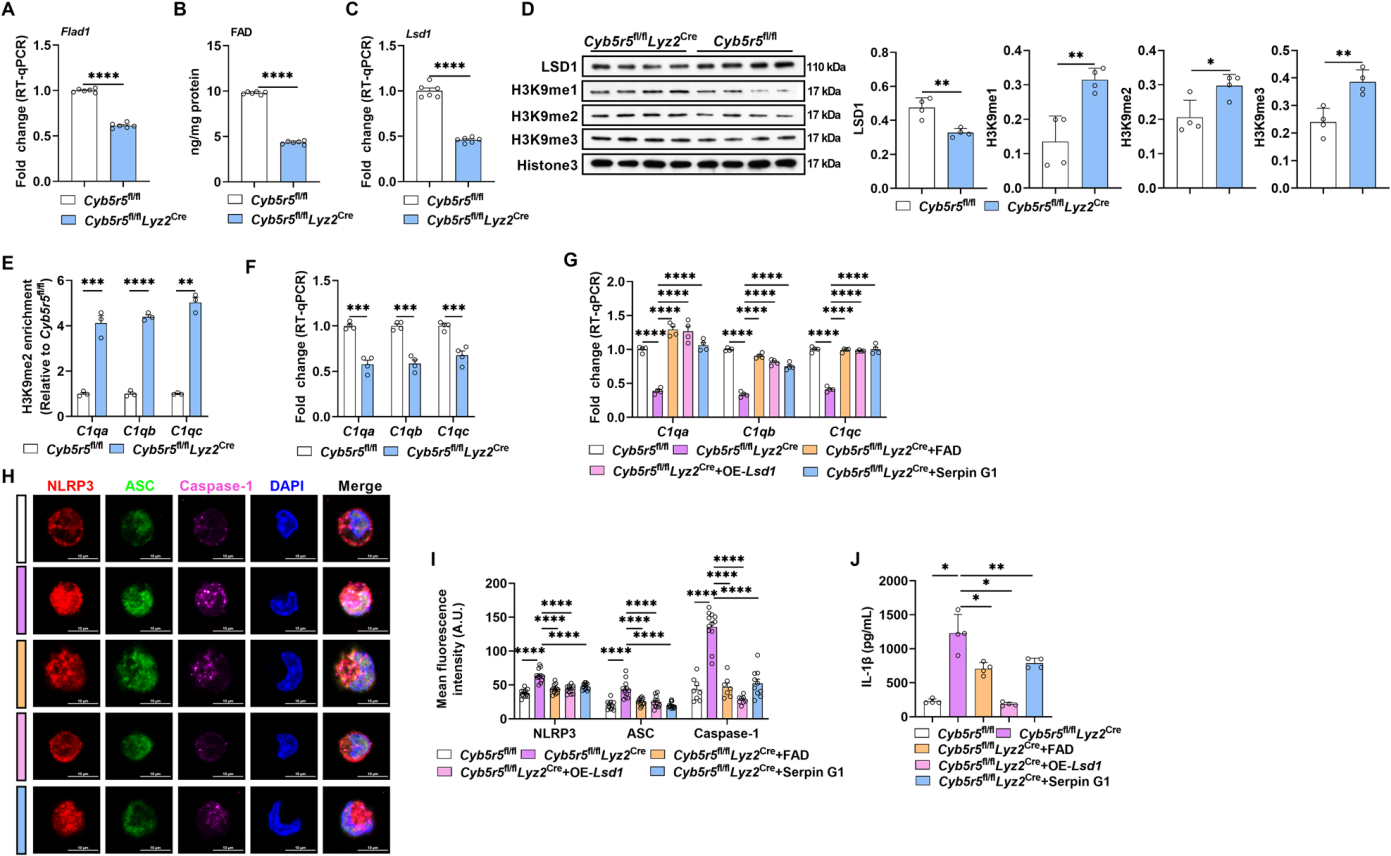
Myeloid depletion of *Cyb5r5* promotes M1 macrophage polarization via FAD-LSD1-C1q axis. **A** Relative mRNA expression of *Flad1* in M1 macrophages from *Cyb5r5*^fl/fl^ and *Cyb5r5*^fl/fl^ *Lyz2*^Cre^ mice (n = 6). **B** The levels of FAD in M1 macrophages from *Cyb5r5*^fl/fl^ and *Cyb5r5*^fl/fl^ *Lyz2*^Cre^ mice (n = 6). **C** Relative mRNA expression of *Lsd1* in M1 macrophages from *Cyb5r5*^fl/fl^ and *Cyb5r5*^fl/fl^ *Lyz2*^Cre^ mice (n = 6). **D** The protein abundance of LSD1, H3K9me1, H3K9me2 and H3K9me3 from M1 macrophages from *Cyb5r5*^fl/fl^ and *Cyb5r5*^fl/fl^ *Lyz2*^Cre^ mice (n = 4). **E** CHIP-qPCR analysis of H3K9me2 enrichment at *C1qa, C1qb*, and *C1qc* (n = 3). **F** Relative mRNA expression of *C1qa, C1qb, and C1qc* in M1 macrophages from *Cyb5r5*^fl/fl^ and *Cyb5r5*^fl/fl^ *Lyz2*^Cre^ mice (n = 4). **G** Relative mRNA expression of *C1qa, C1qb, and C1qc* in M1 macrophages from *Cyb5r5*^fl/fl^ *Lyz2*^Cre^ mice treated with FAD, LSD1 overexpression or Serpin G1 (n = 4). **H**, **I** IF analysis of NLRP3 (red), ASC (green), and Caspase-1 (purple) in M1 macrophages from *Cyb5r5*^fl/fl^ and *Cyb5r5*^fl/fl^ *Lyz2*^Cre^ mice treated as **G** (n = 5 samples and observed 2–3 cells per sample). **J** The secretion of IL-1β from M1 macrophages treated as **G** (n = 4). Data were analyzed with unpaired t-test (**A**–**F**) or one-way ANOVA (**G**, **I**, **J**) and represented with mean ± SD other than RT-qPCR results. *p < 0.05, **p < 0.01, ***p < 0.001, ****p < 0.0001.

To verify the importance of the CYB5R5-FAD-LSD1-C1q signaling pathway in M1 macrophage polarization, we performed RT-qPCR analysis to quantify *Flad1* mRNA levels in CKO M1 macrophages and found *Flad1* was downregulated (Fig. 6A). Notably, myeloid depletion of *Cyb5r5* lowered FAD level in M1 macrophages (Fig. 6B). Myeloid depletion of *Cyb5r5* also resulted in a reduction of mRNA expression level of *Lsd1* (Fig. 6C), concomitant with a decrease in LSD1 protein abundance and an increase in protein abundance of H3K9me1, H3K9me2, and H3K9me3 in M1 macrophages (Fig. 6D). Notably, myeloid depletion of *Cyb5r5* increased H3K9me2 enrichment on C1q-coding genes in M1 macrophages (Fig. 6E). Consequently, myeloid depletion of *Cyb5r5* reduced the expression of C1q-coding genes in M1 macrophages (Fig. 6F). Moreover, supplementation with FAD, LSD1 overexpression, or C1q activation rescued the effects of myeloid depletion of *Cyb5r5* on C1q-coding gene expression (Fig. 6G), and inhibited NLRP3 inflammasome assembly as well as IL-1β production of M1 macrophages (Fig. 6H–J). Collectively, myeloid depletion of *Cyb5r5* promotes M1 macrophage polarization through the FAD-LSD1-C1q signaling pathway.

### CYB5R5 regulates macrophage-associated diseases

**Fig. 7:**
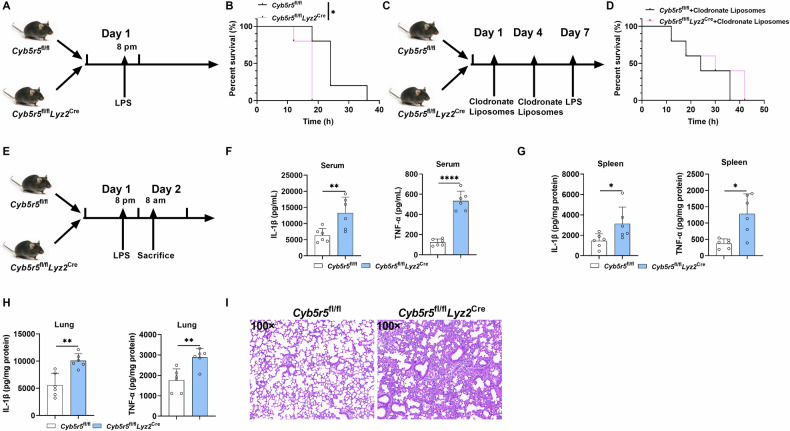
CYB5R5 plays crucial roles in macrophage-associated diseases. **A** Schematic representation of experimental workflow for survival rate of mice treated with LPS (20 mg/kg body weight). **B** Survival rate of mice with myeloid depletion of *Cyb5r5* after LPS (20 mg/kg body weight) challenge (n = 5). **C** Schematic representation of experimental workflow for survival rate of mice treated with clodronate liposomes and then LPS (20 mg/kg body weight). **D** Survival rate of mice with myeloid depletion of *Cyb5r5* treated with clodronate liposomes and then LPS (20 mg/kg body weight) challenge (n = 5). **E** Schematic representation of experimental workflow for sepsis model. **F**–**H** The levels of IL-1β and TNF-α in the serum, spleen, and lung from *Cyb5r5*^fl/fl^ and *Cyb5r5*^fl/fl^ *Lyz2*^Cre^ mice (n = 6). **I** H&E staining (×100) to analyze the lung inflammation from *Cyb5r5*^fl/fl^ and *Cyb5r5*^fl/fl^ *Lyz2*^Cre^ mice (n = 6). Data were analyzed with Gehan-Breslow-Wilcoxon test (**B**, **D**) or unpaired t-test (**F**–**H**) and represented with mean ± SD. *p < 0.05, **p < 0.01, ****p < 0.0001.

We subsequently assessed CYB5R5-mediated anti-inflammatory effects in vivo using LPS-induced sepsis. CKO mice exhibited significantly lower survival rates compared to WT controls (Fig. 7A, B). Notably, macrophage depletion via clodronate liposomes abolished this survival disparity (Fig. 7C, D). CKO mice demonstrated elevated systemic inflammation, with pronounced increases in IL-1β and TNF-α levels across serum, spleen and lung (Figs. 7E–H and S6A, B). Histopathological analysis revealed exacerbated lung hemorrhage in CKO mice (Fig. 7I), corroborating the cytokine profile findings. Collectively, our findings demonstrate that myeloid-specific depletion of *Cyb5r5* exacerbates macrophage-orchestrated inflammation in mice.

## Discussion

Emerging studies demonstrate that CYB5Rs function as essential regulators of intracellular redox homeostasis, orchestrating electron transfer processes that critically influence desaturation of saturated fatty acids and cholesterol biosynthesis [27, 28]. Previous studies show that M1 macrophages have an enhanced fatty acid synthesis [10, 29, 30], implying CYB5R proteins are correlated with M1 macrophage polarization. Interestingly, while the mRNA expression of other CYB5Rs is upregulated, the mRNA expression of CYB5R5 is uniquely downregulated. In this study, we have elucidated the critical function of CYB5R5 in modulating IL-1β production within macrophages. Meanwhile, mechanistic interplay among distinct CYB5R isoforms and their potential functional divergence remain unresolved. Although CYB5Rs share redox-regulatory roles, the unique suppression of CYB5R5 in inflammation and its isoform-specific coupling to the FAD-LSD1-C1q axis suggests non-redundant control over macrophage epigenetics. Future studies are needed to test for compensatory upregulation of CYB5R1-4 in CYB5R5-deficient models. While CYB5R5 modulates M2 polarization, its mechanistic determinants require systematic delineation.

The complement system has historically been recognized to facilitate inflammation and pathogen clearance [31]. Although these functions are indispensable for host defense, recent advancements have revealed an additional role for complement components in defense against inflammatory pathologies [32]. Notably, the complement component C1q is pivotal in directing macrophage polarization, leading to reduced pro-inflammatory cytokines [33, 34]. Consistently, our findings suggest that CYB5R5 modulates C1q expression, thereby influencing the inflammatory responses. Furthermore, other C1q family constituents (e.g., complement mannose-binding lectin, MBL and adiponectin) also exhibit the ability to downregulate macrophage-mediated inflammatory responses [35]. Future studies are needed to determine whether CYB5R5 deficiency dysregulates anaphylatoxins (e.g., C3a/C5a), potentially amplifying inflammatory cascades in sepsis. Therefore, whether other complement components are involved in CYB5R5-mediated inflammatory responses requires more investigations.

Macrophage polarization requires transcriptional precision orchestrated through multifactorial coordination, particularly transcriptional regulators and epigenetic reprogramming [36]. Epigenetic modifications are predominantly divided into three fundamental epigenetic mechanisms: histone modifications, DNA methylation, and ncRNAs. Among these, histone lysine demethylases (KDMs) critically regulate chromatin transcription by modulating methylation status of histone H3 and H4 at lysine residues. Typically, methylation of H3 at K4, K36, and K79 is correlated with transcriptional activation, whereas others are correlated with transcriptional repression [37]. The KDM family comprises two major subfamilies: FAD-dependent KDM1 (LSD1) and α-ketoglutarate-dependent KDMs (KDM2-7), which collectively govern macrophage functional plasticity by mediating site-specific histone demethylation and chromatin remodeling [38]. In this study, we demonstrated that CYB5R5 overexpression activates FAD-LSD1 signaling pathway. While we established the pivotal regulatory axis of LSD1 in CYB5R5-driven macrophage polarization through pharmacological and genetic inactivation, lineage-restricted genetic models (e.g., *Lsd1*^fl/fl^*Lyz2*^cre^) remain warranted to validate cell-autonomous regulatory mechanisms.

The observation that myeloid depletion of *Cyb5r5* promotes M1 macrophage polarization through the FAD-LSD1-C1q signaling pathway reveals pathophysiological significance in macrophage-associated diseases. Macrophages critically regulate the pathogenesis of various diseases, including sepsis, rheumatoid arthritis, and atherosclerosis [39]. Here we observed that CYB5R5 deficiency significantly worsens sepsis in murine models. The *Lyz2*^Cre^ model, though commonly employed for macrophage-specific interrogation, demonstrates promiscuous recombination across myeloid-derived populations (monocytes, granulocytes, CD11C^+^ DCs), resulting in concomitant gene ablation [40]. The functional characterization of CYB5R5 deficiency requires systematic interrogation, particularly regarding its isoform-specific effects within the CYB5R redox enzyme family.

Collectively, we establish CYB5R5’s central regulatory role in M1 polarization through FAD-dependent LSD1 axis (Fig. S7). This cascade attenuates NLRP3 inflammasome assembly, and CYB5R5 deficiency exacerbates LPS-induced septic pathology, positioning it as a potential immunometabolic checkpoint for macrophage-driven disorders.

## Materials and methods

### Mice

The *Cyb5r5*^fl/fl^ and *Lyz2*^Cre^ murine strains, both maintained on a C57BL/6 J genetic background, were developed through genetic engineering protocols at Cyagen Biosciences Inc. (Guangzhou, China). This study utilized these genetically modified mouse models to produce *Cyb5r5*^fl/fl^*Lyz2*^Cre^ mice. Protocols were sanctioned by the Laboratory Animal Ethical Commission of South China Agricultural University, with the authorization number: 2024f337. Each experimental cohort consisted of mice that were age- and sex-matched to ensure uniformity across groups.

### Cell lines

THP-1 cells were cultured in complete RPMI 1640 medium and treated with 100 nM PMA for 48 hours. 3D4/21 and ANA.1 cells were cultured in complete DMEM. All complete medium was supplemented with 10% FBS and 1% penicillin-streptomycin. Subsequently, the cells were treated as indicated.

### Peritoneal macrophages

Peritoneal macrophages (PEMs) were obtained in mice following a 3-day incubation post-administration of 4% thioglycolate. Subsequently, macrophages were retrieved from the peritoneal cavity using DMEM. Upon 2 hours adherence-based purification, the macrophages were exposed to complete DMEM medium treated as indicated.

### Bone marrow-derived macrophages

Mouse femur and tibia bones were dissected, and the marrow cavity was exposed. Then bone marrow was extracted using ice-cold RPMI 1640 medium and centrifugated at 300 × *g* for 3 minutes. Cell pellet was subsequently re-suspended using RBC lysis buffer. The rest were then resuspended with complete RPMI 1640 medium containing MCSF (20 ng/ml). Post a 7-day cultivation, the cells were subjected to the indicated treatments.

### Dual-luciferase reporter assays

293 T cells were seeded in 96-well plates and incubated overnight. Cells were transfected with the wild type *Cyb5r5* reporter plasmid or mutant *Cyb5r5* plasmid and p65 plasmid using Lipofectamine 3000 reagent for 36 h. The Promega Dual-Luciferase® Reporter Assay System was used to measure firefly luciferase activity, normalized to renilla luciferase activity as an internal control.

### Cytokine measurement

Cytokines were quantitatively analyzed utilizing commercial ELISA kits in strict adherence to the manufacturer’s standardized protocols. For cellular supernatant preparation, the samples were collected and subjected to low-speed centrifugation at 1200 rpm for 5 minutes to remove particulate matter before analytical processing. Tissue samples underwent mechanical homogenization and centrifugation at 14,000 rpm for 5 minutes at 4 °C, while serum was similarly processed through high-speed centrifugation under identical parameters to separate cellular components prior to cytokine quantification.

### FAD measurement

FAD levels in macrophages were quantified using the FAD Assay Kit in strict adherence to standard protocols. Cell samples were homogenized in FAD Assay Buffer and centrifuged to collect supernatant. Then samples were deproteinized using methanol and acetonitrile (1:1, v/v) and resuspended in FAD Assay Buffer. Last, samples were subjected to kinetic analysis by stoichiometrically combining 50 μL aliquots with reaction cocktail containing specific FAD-converting enzymes. Following light-protected incubation at 25 °C for 30 minutes, spectrophotometric quantification was performed at 570 nm absorbance with parallel blank subtraction.

### Seahorse assay

Cellular bioenergetic profiling of macrophages was performed through real-time metabolic flux analysis under standardized experimental conditions. Cells were equilibrated in non-buffered XF assay medium in strict adherence to standardized protocols to establish baseline metabolic parameters. Mitochondrial respiratory function was interrogated through sequential pharmacological modulation: 100 μM oligomycin, 100 μM carbonyl cyanide 4-(trifluoromethoxy) phenylhydrazone (FCCP), and 50 μM rotenone/antimycin A to determine non-mitochondrial oxygen consumption. Parallel glycolytic profiling was conducted via extracellular acidification rate (ECAR) quantification under substrate-constrained conditions, involving sequential administration of 1 mM glucose, 1 mM oligomycin, and 1 mM 2-deoxyglucose. Oxygen consumption rate (OCR) and ECAR were acquired at 8-min intervals following 3-min mixing/2-min equilibration cycles, with data processing performed using Wave Desktop Software incorporating instrument-specific background correction algorithms.

### Western blot

Cell samples were lysed with RIPA buffer. Then the protein concentration was determined using BCA protein assay kits. The protein samples were subsequently separated on SDS-PAGE gels and transferred onto a NC membrane. The membrane was blocked with 5% defatted milk. The membranes were then incubated sequentially with primary antibodies and proper secondary antibodies. Last, protein bands were detected through enhanced chemiluminescence imaging methodology.

### RT-qPCR

This part was conducted by employing the EZ-press RNA Purification Kit following standard protocol. Subsequently, RNA was reverse transcribed into complementary DNA utilizing the EZBioscience 4 × Reverse Transcription Master Mix according to standard instructions. qPCR was conducted with SYBR Green-based fluorescent dye on a QuantStudio 6 Real-Time PCR System. Relative quantification of target gene expression was calculated via the comparative threshold cycle (2^−ΔΔCt^) method.

### Immunofluorescence

The assay was performed following established protocols. Fixed and permeabilized cellular specimens were subjected to blocking with QuickBlock™ Immunostaining Blocking Buffer, incubated with species-specific primary and fluorophore-conjugated secondary antibodies. Subcellular localization of target antigens was examined utilizing confocal fluorescence microscope, with image acquisition parameters standardized across experimental replicates.

### Gene silencing or overexpression

Genetic modulation through targeted gene silencing or overexpression was experimentally induced in cultured macrophages via Lipofectamine 3000-mediated transfection methodology, executed under standardized conditions in accordance with manufacturer specifications. Transfection efficiency and protocol fidelity were ensured through rigorous optimization of lipid-to-nucleic acid ratios and post-transfection viability assays, maintaining experimental reproducibility across biological replicates.

### RNA-seq

Following sample preparation, high-throughput sequencing was conducted in NovoGene Co., Ltd. (Beijing, China) under standardized workflows. Raw sequencing data underwent stringent quality filtering to eliminate low-confidence reads (Phred score <30, adapter contamination, or ambiguous bases), yielding high-quality paired-end reads for downstream analysis. DEGs were selected in accordance with the standards of |log2 fold change | ≥ 1 and *p* < 0.05 using DESeq2 software. The raw sequence data are available in the Genome Sequence Archive (GSA: CRA021778) at China National Center for Bioinformation (https://ngdc.cncb.ac.cn/gsa).

### CHIP-qPCR

Chromatin-protein complexes were crosslinked in situ via formaldehyde treatment (1% final concentration, 10 min at 37 °C) and subsequently fragmented by sonication to generate DNA fragments within a 200-800 bp size range, as verified by agarose gel electrophoresis. Antibody-specific immunoprecipitation was conducted overnight at 4 °C using H3K9me2 antibodies, following magnetic bead-based capture of immune complexes. Post-wash purification of ChIP-enriched DNA was performed via column-based extraction, and quantitative analysis of target loci occupancy was assessed by SYBR Green-based qPCR (ChIP-qPCR) on a QuantStudio 6 system, normalized to input DNA controls.

### Mitochondrial biomass

Mitochondrial biomass quantification was performed using fluorescent probe-based imaging. Post-treatment, cellular specimens were subjected to incubation with 100 nM MitoTracker Red CMXRos dissolved in pre-warmed (37 °C) serum-free medium for 30 minutes under physiological culture conditions (37 °C, 5% CO₂). Unbound dye was removed via three washes with phosphate-buffered saline (PBS). Fluorescence intensity was read at wavelengths 579/599 nm.

### In vivo LPS challenge

Experimental sepsis induction was performed through intraperitoneal administration of LPS (20 mg/kg body weight) dissolved in PBS. Following a 12-hour observation period, subjects were humanely euthanized in accordance with institutional animal care protocols, with subsequent systematic collection of biological specimens including serum, jejunum, colon, spleen, and pulmonary tissue. Longitudinal survival analysis was conducted through a continuous monitoring regimen (0–72 hours post-LPS challenge), with mortality events recorded at 6-hour intervals across all experimental cohorts to ensure temporal resolution in survival curve generation.

### Macrophage depletion

In summary, following complete anesthesia, mice were intraperitoneally administered 200 μL clodronate-containing liposomes twice a week. Then the mortality events were recorded after LPS challenge (20 mg/kg body weight) across all groups.

### H&E analysis

Samples were surgically excised and immersion-fixed in 4% paraformaldehyde to preserve cellular architecture, followed by systematic processing through a standard histological processing pipeline. Sequential dehydration was performed using ascending ethanol concentrations (70–100%), after which tissues were mounted on glass slides. Histomorphology evaluation was conducted via histochemical staining with H&E under standardized protocols, enabling comprehensive microscopic analysis of tissue integrity and pathological alterations.

### Statistical analysis

Quantitative data were represented as mean ± SEM or SD derived from the specified sample sizes, and statistical analyses were conducted using GraphPad Prism software (version 8.0). Individual data points were graphically represented in figures, and precise sample sizes (n) were enumerated within the figure legends. A minimum of three biological replicates were employed for all experimental procedures. Survival rates were evaluated via the Gehan-Breslow-Wilcoxon test. For parametric comparisons, the unpaired Student’s t-test was applied to datasets demonstrating normal distribution (assessed by the D’Agostino–Pearson omnibus or Kolmogorov–Smirnov tests) and homogeneity of variance (verified by F-test or Brown–Forsythe test), with Welch’s correction implemented for unequal variances. Nonparametric datasets were analyzed using the Mann–Whitney U test for two-group comparisons. Multi-group analyses of normally distributed data with equal variance were performed using one-way ANOVA followed by Dunnett’s post hoc test, while non-normally distributed multi-group data were assessed via the Kruskal-Wallis test with Dunn’s multiple comparisons correction. Statistical significance was defined as a two-tailed *p* < 0.05 for all analyses. The experimental design did not incorporate investigator blinding for allocation, animal handling, or outcome assessment. Furthermore, animal studies and those employing animal-derived cells/tissues were conducted without randomization. Sample sizes were selected empirically, informed by historical data on experimental variability, and were not predetermined using formal statistical power calculations.

## Supplementary information

supplementary information

Reproducibility checklist

Original western blots

## Data Availability

The datasets supporting the findings of this study are available from the corresponding author upon reasonable request.
