# Supplementary material for: Cytochrome b5 reductase orchestrates IL-1β production in macrophages through FAD
Source: Cell Death Dis. 2025 Oct 21;16(1):742. doi: 10.1038/s41419-025-08073-2 (PMC12540738; doi:10.1038/s41419-025-08073-2)
Supplement: Supplementary file 1 — supplementary information [file 41419_2025_8073_MOESM1_ESM.docx]

**Cytochrome b5 reductase orchestrates IL-1β production in macrophages through FAD**

Jian Fu, Zhihua Liu, Hangchao Zhang, Xinmei Zhang, Shijie Liu, Xuehua Mei, Xiu Zeng, Wenkai Ren

**Table. 1 Key resource in the study**

| **REAGENT or RESOURCE** | **SOURCE** | **IDENTIFIER** |
| --- | --- | --- |
| **Antibodies** |  |  |
| CYB5RL | Themo Fisher | PA5-70199 |
| p65 | Proteintech | 10745-1-AP |
| p-p65  p-p105 | CST  Abcam | #3033S  ab194729 |
| mTOR | CST | 2972s |
| p-mTOR | CST | 5536s |
| STAT6 | Abcam | ab32520 |
| p-STAT6 | Abcam | ab235591 |
| IL-1β | Santa Cruz | Sc-7884 |
| NLRP3 | Abcam | ab214185 |
| Caspase-1 | Abcam | ab179515 |
| DRP1 | Abcam | ab156951 |
| OPA1 | Proteintech | 27733-1-AP |
| MFN1 | Proteintech | 13798-1-AP |
| MFN2 | Proteintech | 12186-1-AP |
| LSD1 | Abcam | ab129195 |
| H3K9me1 | Abcam | ab176880 |
| H3K9me2 | Abcam | ab32521 |
| H3K9me3 | Abcam | ab176916 |
| ARG1 | Abcam | 16001-1-AP |
| CD206 | Proteintech | 18704-1-AP |
| Histone3 | Abcam | ab176840 |
| β-Actin | Proteintech | 66009-1-Ig |
| NLRP3 for immunofluorescence | Abcam | ab4207 |
| ASC for immunofluorescence | Santa Cruz | SC-365611 |
| Caspase-1 for immunofluorescence | Proteintech | 22915-1-AP |
| **Chemicals** |  |  |
| Lipopolysaccharide (LPS) | Sigma | L2880 |
| Recombinant murine IFN-γ | PeproTech | 315-05-20 |
| Recombinant murine IL-4  Recombinant murine M-CSF | PeproTech  PeproTech | 214-14  315-02 |
| Clodronate Liposomes | YEASEN | 40337ES08 |
| Thioglycollate medium | Sigma | 70157 |
| Lipofectamine™ 3000  IKK16 | Invitrogen  MCE | L3000015  HY-13687 |
| FAD  Serpin G1  GSK2879552  DAPI | MACKLIN  MCE  MCE  Beyotime | F875772  HY-P71297  HY-18632  C1005 |
| MCC950 | MCE | HY-12815 |
| VX765 | MCE | HY-13205 |
| **Critical Commercial Assays** |  |  |
| IL-1β ELISA | Proteintech | KE10003 |
| TNF-α ELISA | Proteintech | KE10002 |
| Seahorse XF Cell Mito Stress Test Kit | Agilent | 103015-100 |
| Seahorse XF Glycolysis Stress Test Kit | Agilent | 103020-100 |
| FAD detection kit  One Step Mouse Genotyping Kit  Dual-Glo® Luciferase Assay System  SimpleChIP® Enzymatic Chromatin IP Kit | Abcam  Vazyme  Promega  CST | ab204710  PD101-01  E2920  9002S |
| **Deposited Data** |  |  |
| RNA-seq | CNCB | CRA021778 |
| **Cell Lines** |  |  |
| 3D4/21  293T  ANA.1 | ATCC  ATCC  Yangzhou Universiy | N/A  N/A  N/A |
| THP-1 | Tongji University | N/A |
| **Oligonucleotides** |  |  |
| siRNA for murine *Cyb5r5*  FW: GGAACGUGCAAACCUUCUU  RW: AAGAAGGUUUGCACGUUCC  siRNA for murine *C1qa*  FW: UUGGCAACGUGGUUAUCUUUGACAA | Gene create  Gene create | N/A  N/A |
| RW: UUGUCAAAGAUAACCACGUUGCCAA |  |  |
| siRNA for murine *C1qb* | Gene create | N/A |
| FW: UGAUCACCAACGCGAACGAGAACUA  RW: UAGUUCUCGUUCGCGUUGGUGAUCA |  |  |
| siRNA for murine *C1qc* | Gene create | N/A |
| FW: ACAGAAGCACCAGUCGGUAUUCACA  RW: UAUGAAUACCGACUGGUGCUUCUCU |  |  |
| siRNA for murine *Flad1* | Gene create | N/A |
| FW: GCUGGUCUCUGUCCGAAAU  RW: AUUUCGGACAGAGACCAGC |  |  |
| siRNA for murine *Lsd1* | Ribobio | N/A |
| FW: CGGAAGAAGAAAGAAAUGC  RW: GCAUUUCUUUCUUCUUCCG |  |  |
| **Primers for RT-qPCR**  *Cyb5r5* FW: GACGGAGGAGGAGGAGGATTCTG  RW: TTCCCATCTTTCCAGGTCTCGGTAG | Sangon Biotech | N/A |
| pig *CYB5R5* FW: GTCCGCACCTTCTTTGTCCTCAG  RW: ACCAAGGCAAACGGCTTTCTCC | Sangon Biotech | N/A |
| human *CYB5R5* FW: TACCGTGTCCGGTTTGCTCTA  RW: ACTATCCCTCGTAGGATGAGGT | Sangon Biotech | N/A |
| *C1qa* FW: GGACTGGTATCCGAGGTTTTAA  RW: GATATTGCCTGGATTGCCTTTC | Sangon Biotech | N/A |
| *C1qb* FW: CTACACAGAAAGTCGCCTTCTC  RW: CTGGCATGATAGGTGAAGTAGT | Sangon Biotech | N/A |
| *C1qc* FW: TACTTCGTCTACTACACATCGC  RW: GGAAACAGTAGGAAACCAGAGA | Sangon Biotech | N/A |
| *Ccr5* FW: GCTCATCTTTGCCATCATGATT  RW: ATAGATGACAGGGTTTAGGCAG | Sangon Biotech | N/A |
| *Cxcr3* FW: GTGCTAGTGGATATCCTCATGG  RW: AACAACATCCACATTTGCTCTC | Sangon Biotech | N/A |
| *Cxcl9* FW: AATCCCTCAAAGACCTCAAACA  RW: TCCCATTCTTTCATCAGCTTCT | Sangon Biotech | N/A |
| *Lck* FW: AGGGAGAAGTGGTGAAACATTA  RW: AATCCGGGAAAAGTGATACGAG | Sangon Biotech | N/A |
| *Flad1* FW: TCCTGATGTTCCAAAACCTCTCC  RW: CTCGGCTTCTAACACCTGGAGA | Sangon Biotech | N/A |
| *Lsd1* FW: GTGGTGTTATGCTTTGACCGT  RW: GCTGCCAAAAATCCCTTTGAGA | Sangon Biotech | N/A |
| *Actb* FW: GGCTGTATTCCCCTCCATCG  RW: CCAGTTGGTAACAATGCCATGT | Sangon Biotech | N/A |
| pig *ACTB* FW: GATTACTGCTCTGGCTCCTAGC  RW: GCTCAGGAGGAGCAATGATCTT | Sangon Biotech | N/A |
| human *ACTB* FW: CATGTACGTTGCTATCCAGGC  RW: CTCCTTAATGTCACGCACGAT | Sangon Biotech | N/A |
| H3K9me2 CHIP Primers 1 for *C1qa* FW:  GCTGTGTCAAGACCCACTGTGTAC  RW: TGTCCTAACAAGAGAGCCGTCCAG | Sangon Biotech | N/A |
| H3K9me2 CHIP Primers 2 for *C1qb* FW:  CTGTGGAAGGTCCCCTGGAACTAG  RW: GGGGCTGGAGAGATGGCTCAG | Sangon Biotech | N/A |
| H3K9me2 CHIP Primers 3 for mouse *C1qc* FW:  AGTGGCCCGTGTGCTCTCC  RW: CGTGCGTGAATGAAGACTCTACCC  **Primers for genotype**  *Cyb5r5* flox LoxP-1  FW: TGTTCATACCTCATAGCTGTCATC  RW: ACCGATGCTGTGAGCAGTCTG  Lyz2-cre  FW: CTTGGGCTGCCAGAATTTCTC  RW: CCCAGAAATGCCAGATTACG  **Recombinant DNA**  Plasmid: pcDNA3.1-*Cyb5r5* (wild type)  Plasmid: pcDNA3.1-*Lsd1* (wild type)  Plasmid: pPRO-RB-report-*Cyb5r5* (wild type)  Plasmid: pPRO-RB-report-*Cyb5r5* (mutant)  Plasmid: pcDNA3.1-p65 | Sango Biotech  Sangon Biotech  Sangon Biotech  Gene create  Gene create  Gene create  Gene create  Gene create | N/A  N/A  N/A  N/A  N/A  N/A  N/A  N/A |

| **Software** |  |  |
| --- | --- | --- |
| Wave Software Version 2.3 | Agilent | www.agilent.com |
| ImageJ | NIH | www.imagej.nih.gov |
| Graphpad Prism 8 | GraphPad Software | www.graphpad.com |
| ZEN 3.2 | Zeiss | www.zeiss.com |
| QuantStudioTM Real-Time PCR V1.7.2 | QuantStudio™ Real-Time PCR Software | Applied biosystems by Thermo Fisher |

**FIGURE LEGENDS**

**Supplementary figure 1 (Related to Figure 1).
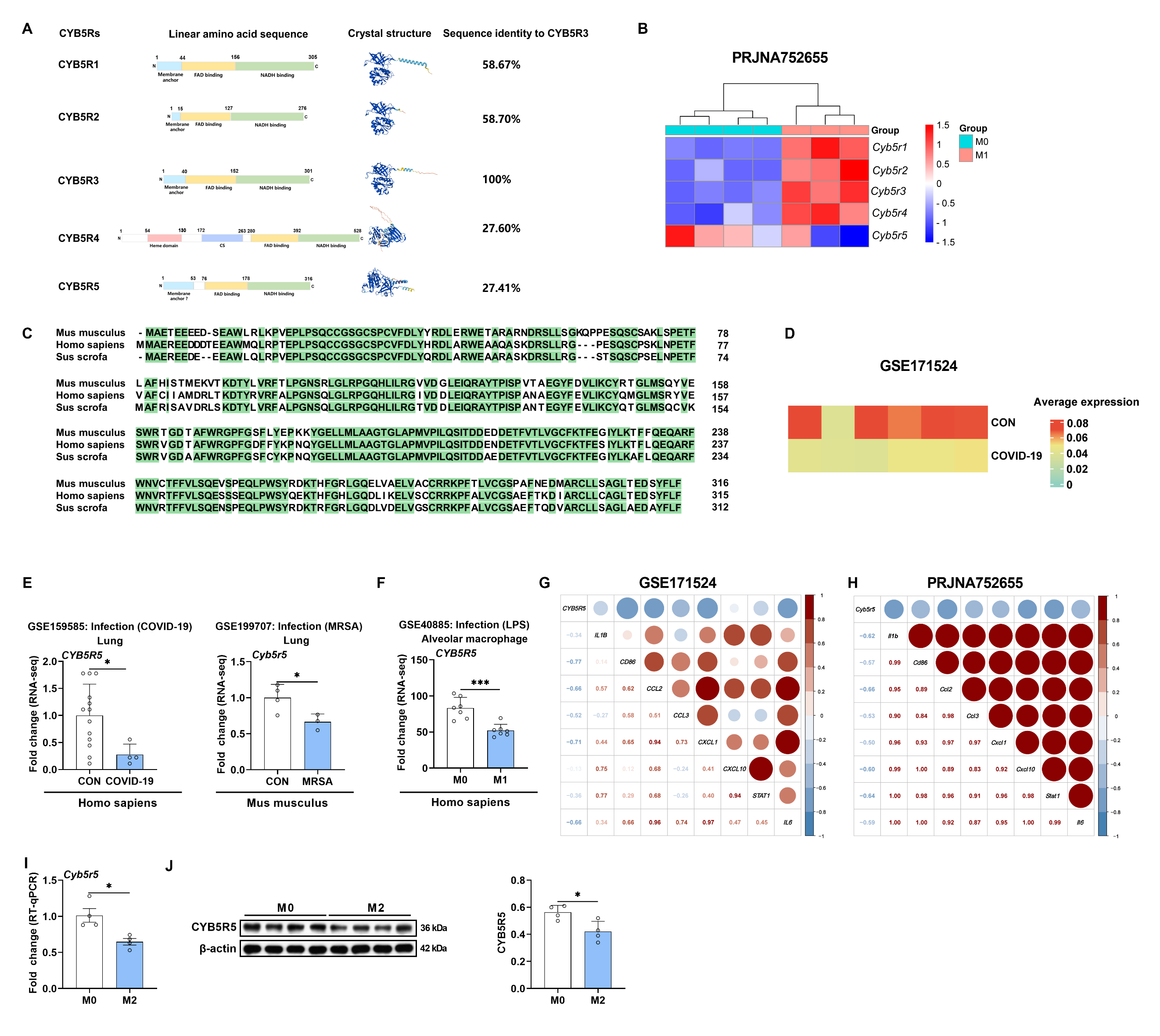
**

(A) Linear amino acid sequence and crystal structure of CYB5R1-5. (B) Heatmap analysis of genes of CYB5R1-5 in M1 macrophages (n=3-4). (C) The amino acid sequence similarity of CYB5R5 among different species. (D) Heatmap analysis of genes of CYB5R5 in alveolar macrophages of COVID-19 patients (n=6). (E) Relative mRNA expression of *Cyb5r5* in lung of human (left, n=4-13) and mouse (right, n=3-4) under infectious conditions. (F) Relative mRNA expression of *Cyb5r5* in human alveolar macrophages (n=7). (G-H) Pearson correlation between *Cyb5r5* and M1 makers including IL-1β, CD86, CCL2, CCL3, CXCL1, CXCL10, STAT1 and IL-6. (I) Relative mRNA expression of *Cyb5r5* in M2 macrophages (n=4). (J) The protein abundance of CYB5R5 in M2 macrophages (n = 4). Data were analyzed with unpaired t-test (D-F, I, J) and represented with mean±SD other than RT-qPCR results. **p* < 0.05.

**Supplementary figure 2 (Related to Figure 2).
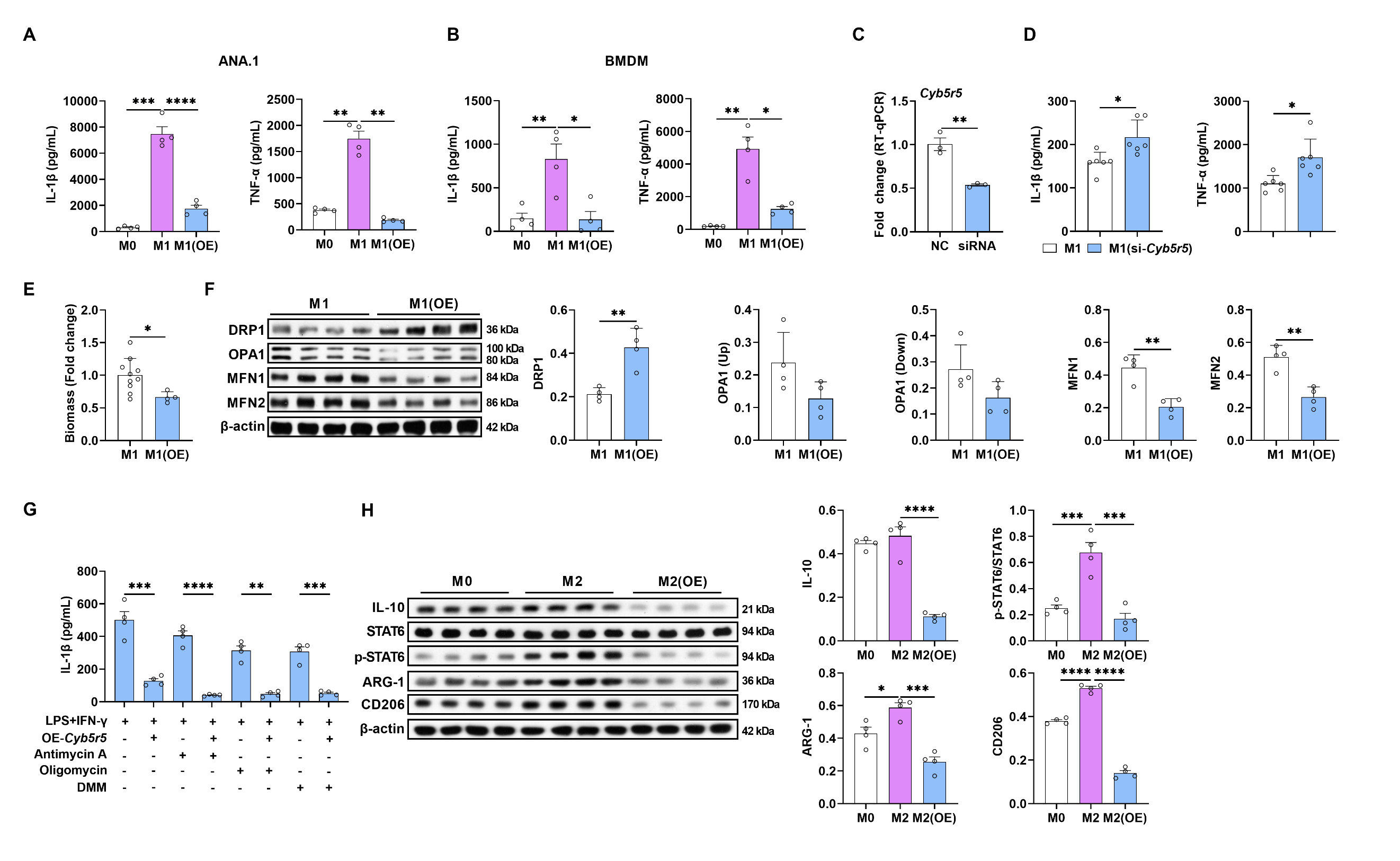
**

(A) The secretion of IL-1β and TNF-α from ANA.1 treated with CYB5R5 overexpression (n = 4). (B) The secretion of IL-1β and TNF-α from BMDM treated with CYB5R5 overexpression (n = 4). (C) Relative mRNA expression of *Cyb5r5* in macrophages transfected with *Cyb5r5* siRNA (n=3). (D) The secretion of IL-1β and TNF-α from macrophages transfected with *Cyb5r5* siRNA (n=6). (E) The biomass of M1 macrophages treated with CYB5R5 overexpression (n=4-10). (F) The protein abundance of DRP1, OPA1, MFN1, and MFN2 in M1 macrophages treated with CYB5R5 overexpression (n = 4). (G) The secretion of IL-1β from PEMs treated with or without CYB5R5 overexpression in the presence of antimycin A, oligomycin or DMM (n = 4). (H) The protein abundance of IL-10, STAT6, p-STAT6, ARG-1 and CD206 in M2 macrophages treated with CYB5R5 overexpression (n = 4). Data were analyzed with unpaired t-test (C-F) or one-way ANOVA (A, B, G, H) and represented with mean±SD other than (C). **p* < 0.05, ***p* < 0.01, ****p* < 0.001, *****p* < 0.0001.

**Supplementary figure 3 (Related to Figure 3).
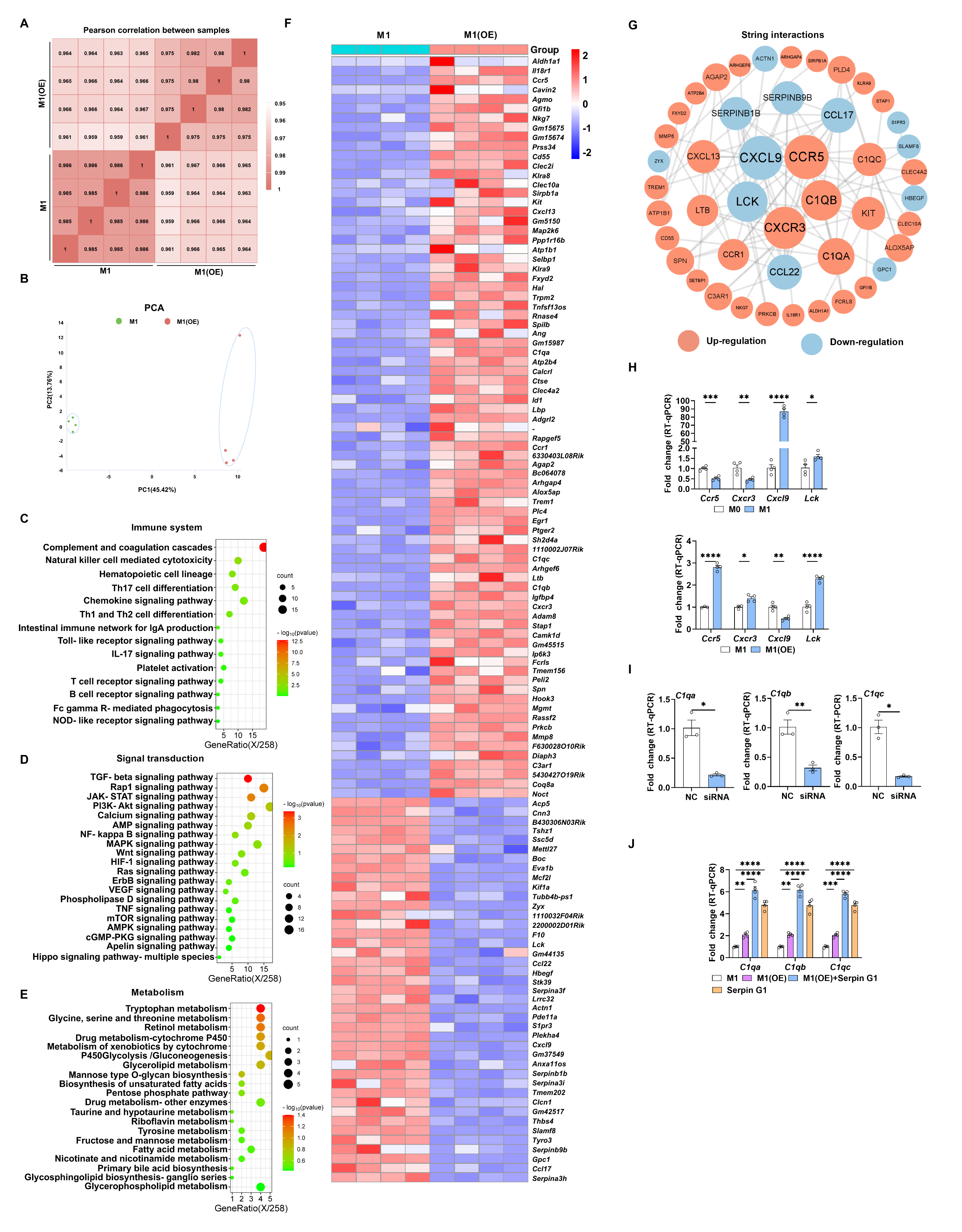
**

(A) Pearson correlation between samples from M1 and M1(OE) groups (n=4). (B) PCA analysis between M1 and M1(OE) groups (n=4). (C-E) Histogram of DEGs in Fig. 3A enriched in immune system (C), signal transduction (D) and metabolism (E). (F) Heatmap analysis of selected 121 key DEGs (n=4). (G) String interactions of proteins coded by key genes in F. (H) Relative mRNA expression of *Ccr5*, *Cxcr3*, *Cxcl9*, and *Lck* in PEMs between M1(OE) vs. M1 and M1 vs. M0 groups. (n = 3-4). (I) Relative mRNA expression of *C1qa, C1qb, and C1qc* in macrophages transfected with corresponding siRNA (n=3). (J) Relative mRNA expression of *C1qa, C1qb, and C1qc* in macrophages treated with CYB5R5 overexpression or CYB5R5 overexpression plus Serpin G1 or Serpin G1 (n=4). Data were analyzed with unpaired t-test (H, I) or one-way ANOVA (J)and represented with mean±SEM. **p* < 0.05, ***p* < 0.01, ****p* < 0.001, *****p* < 0.0001.

**Supplementary figure 4 (Related to Figure 4).
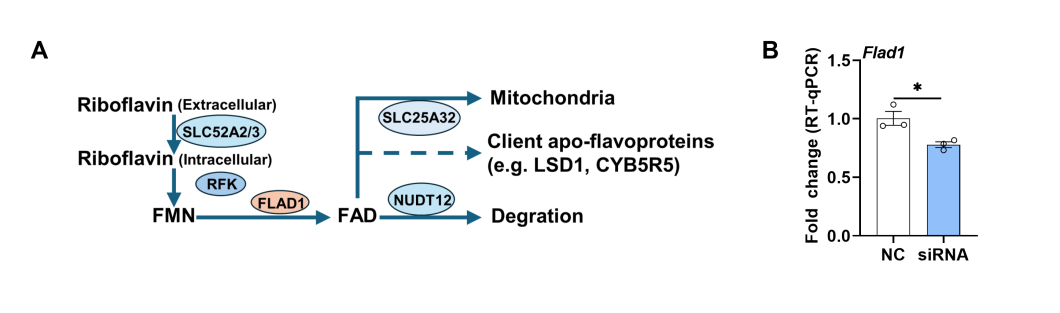
**

(A) FAD biosynthesis, transportation and degradation in macrophages. (B) Relative mRNA expression of *Flad1* in macrophages transfected with *Flad1* siRNA (n=3). Data were analyzed with unpaired t-test (B) and represented with mean±SEM. **p* < 0.05.

**Supplementary figure 5 (Related to Figure 5).
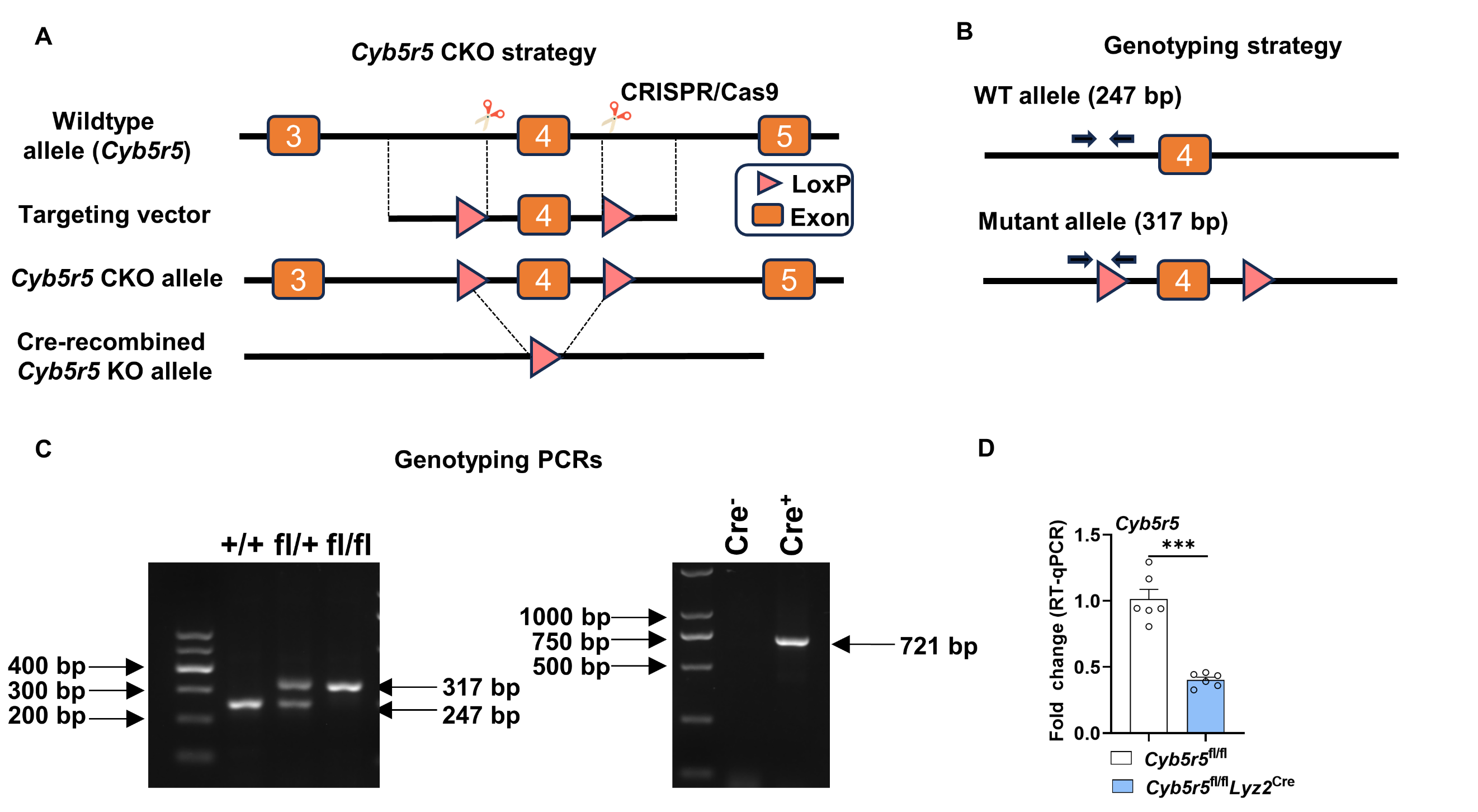
**

(A) The schematic diagram of CRISPR/Cas9 edited of *Cyb5r5* gene. (B) Genotyping strategy of WT allele and mutant allele for *Cyb5r5* CKO. (C) Genotyping PCRs of *Cyb5r5* gene (left panel) and *Lyz2* ^cre^ (right panel)*.* (D) Relative mRNA expression of *Cyb5r5* in macrophages from *Cyb5r5*^fl/fl^ and *Cyb5r5*^fl/fl^ *Lyz2*^Cre^ mice (n = 6). Data were analyzed with unpaired t-test (D) and represented with mean±SEM. ****p* < 0.001

**Supplementary figure 6 (Related to Figure 7).
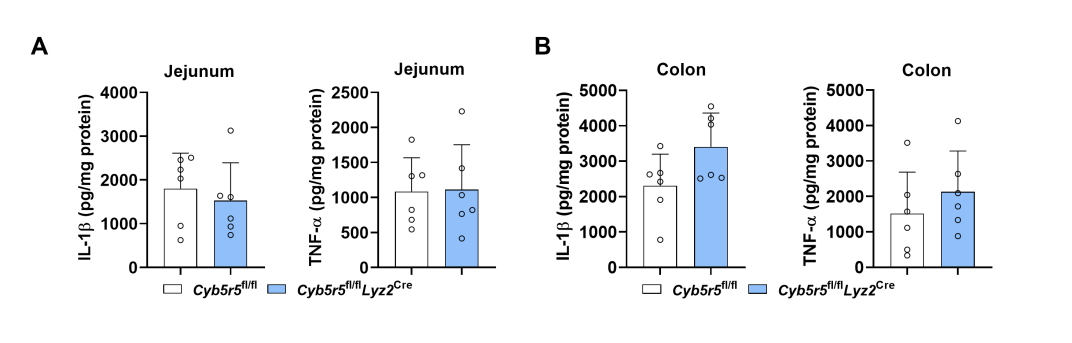
**

(A) The IL-1β and TNF-α levels in the jejunum from *Cyb5r5*^fl/fl^ and *Cyb5r5*^fl/fl^ *Lyz2*^Cre^ mice (n=6). (B) The IL-1β and TNF-α levels in the colon from *Cyb5r5*^fl/fl^ and *Cyb5r5*^fl/fl^ *Lyz2*^Cre^ mice (n=6). Data were analyzed with unpaired t-test (A-B) and represented with mean±SD.

**Supplementary figure 7. CYB5R5 orchestrates M1 polarization through FAD-dependent LSD1 axis.**


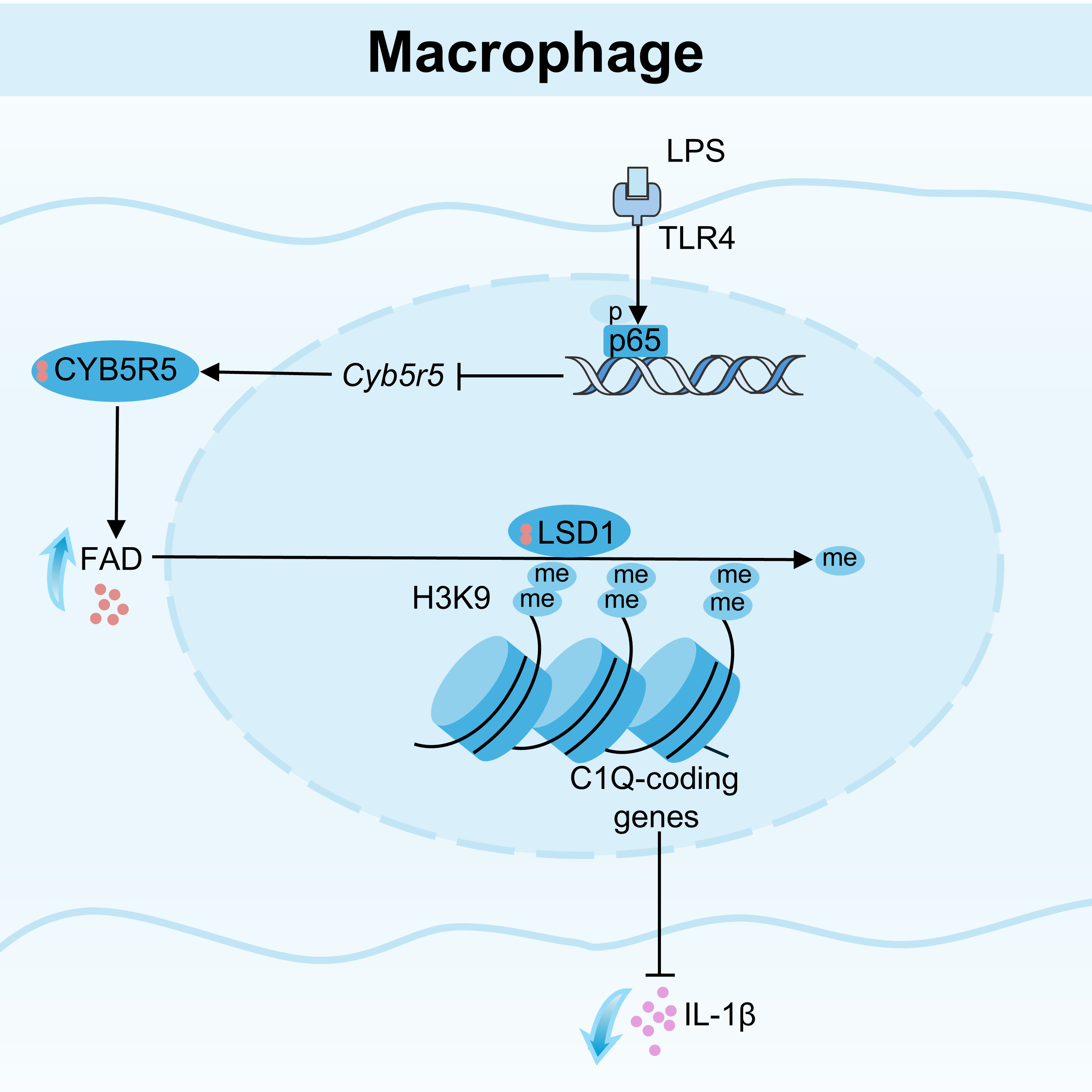


Mechanistically, CYB5R5 enhances FAD-LSD1 signaling to regulate the histone demethylation of C1q-coding genes, thereby lowering NLRP3 inflammasome assembly.
