# Supplementary material for: Cytochrome b5 reductase orchestrates IL-1β production in macrophages through FAD
Source: Cell Death Dis. 2025 Oct 21;16(1):742. doi: 10.1038/s41419-025-08073-2 (PMC12540738; doi:10.1038/s41419-025-08073-2)

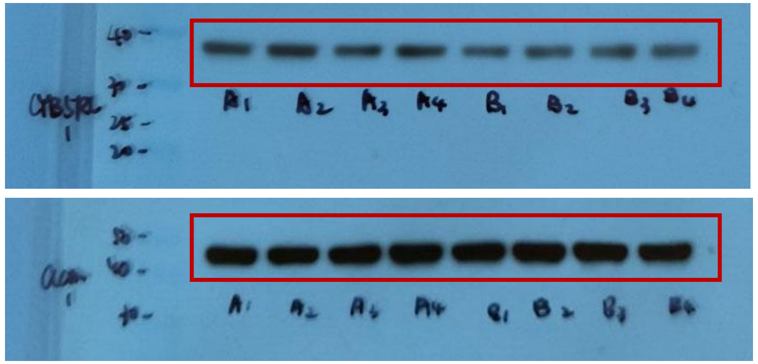

Figure 1B

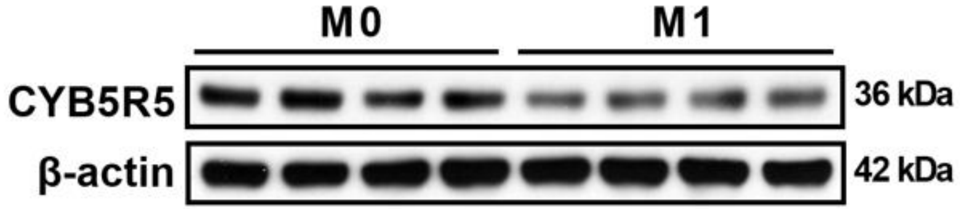

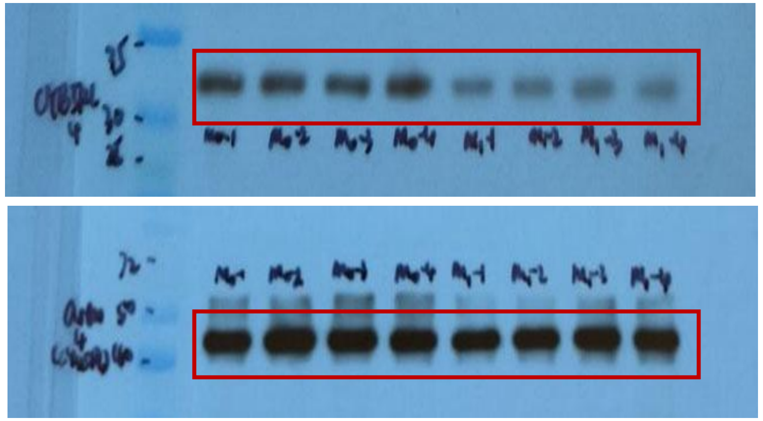

Figure 1D

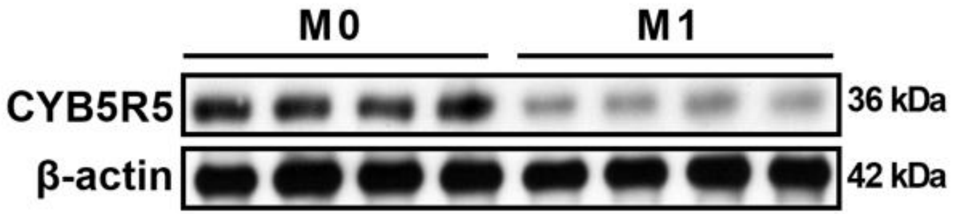

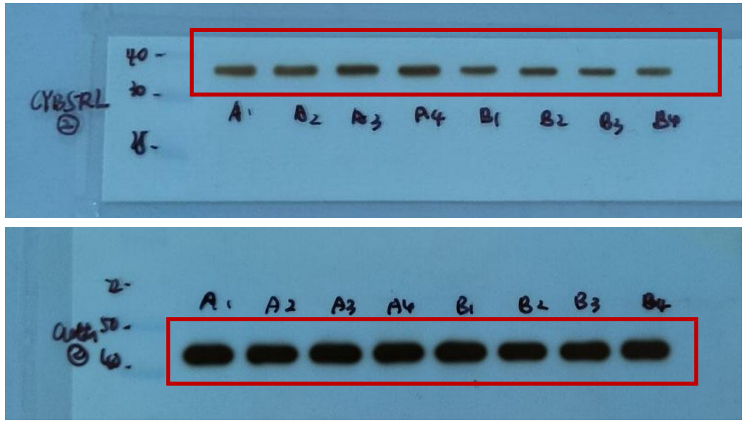

Figure 1F

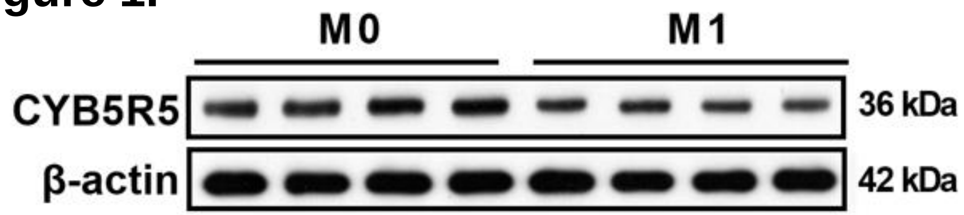

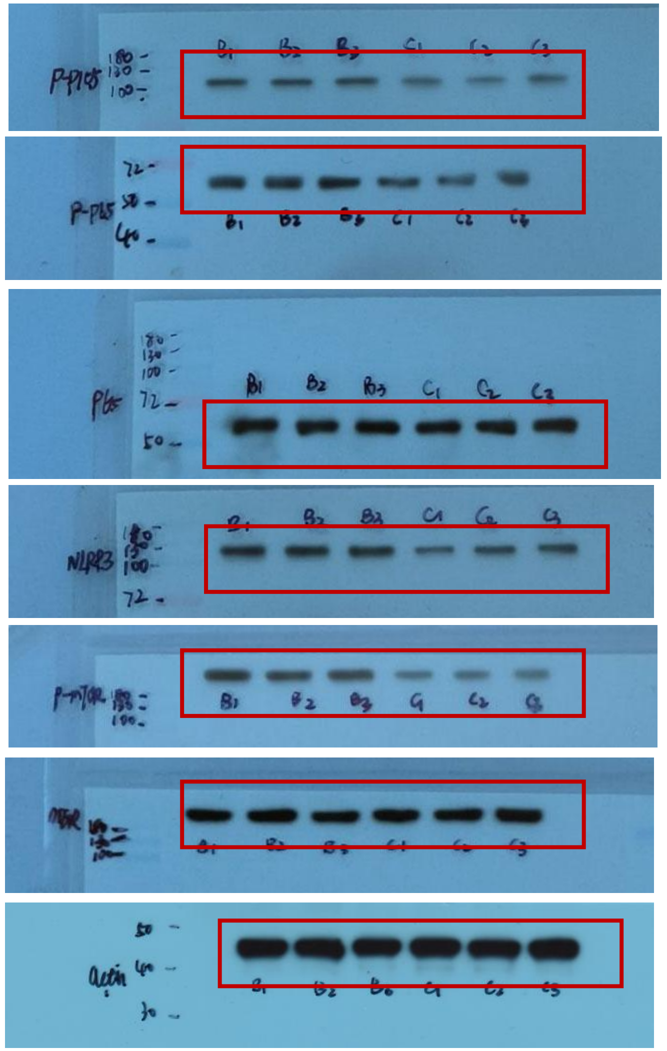

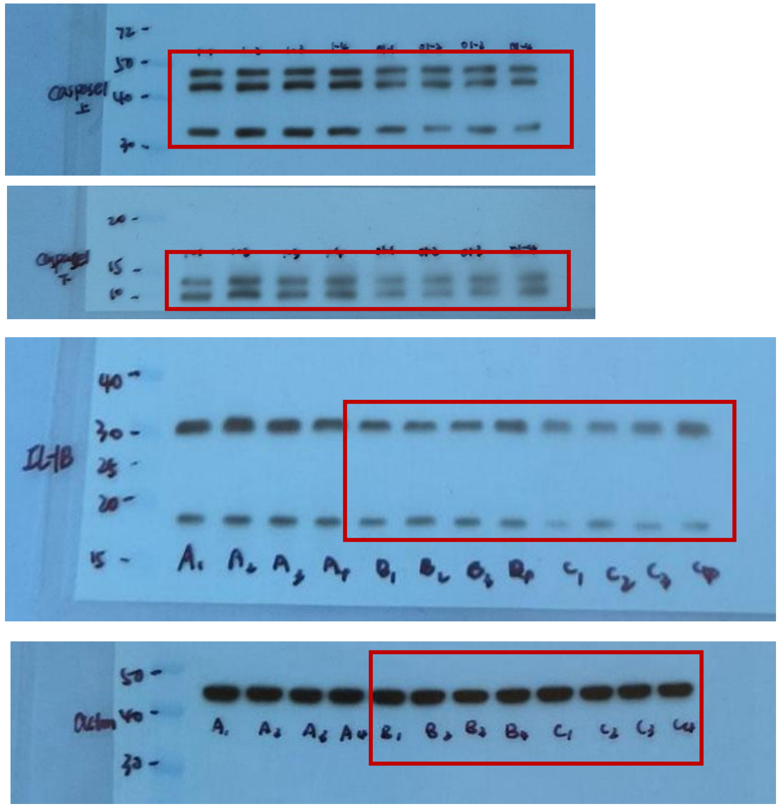

Figure 2E

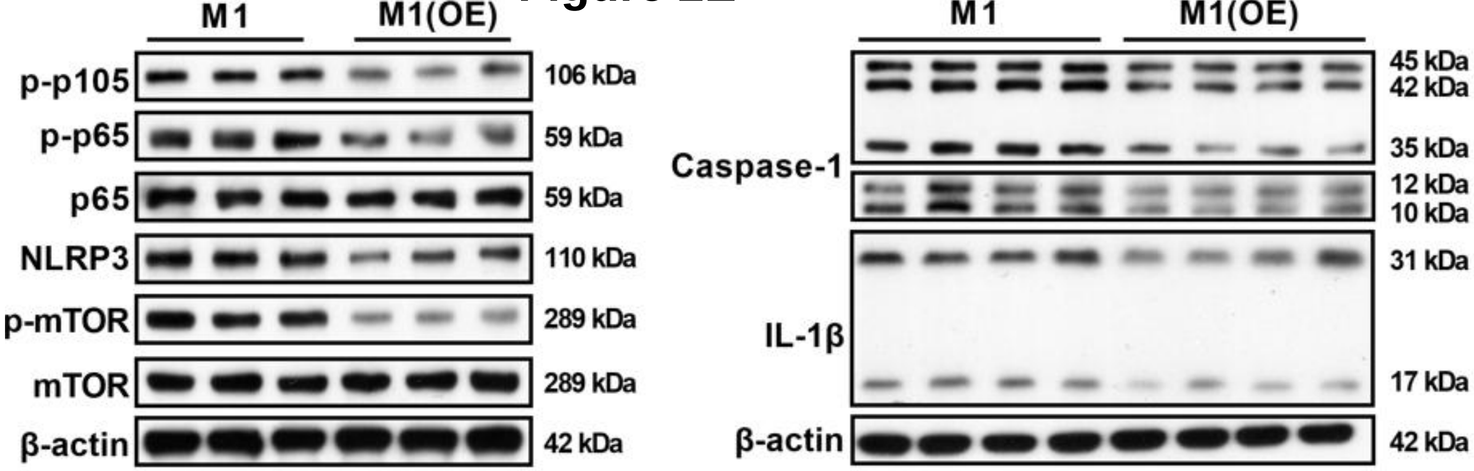

M1
M1(OE)
p-p105
106 kDa
p-p65
59 kDa
p65
59 kDa
NLRP3
110 kDa
p-mTOR
289 kDa
mTOR
289 kDa
β-actin
42 kDa
Caspase-1
45 kDa
42 kDa
35 kDa
12 kDa
10 kDa
IL-1β
31 kDa
17 kDa
β-actin
42 kDa

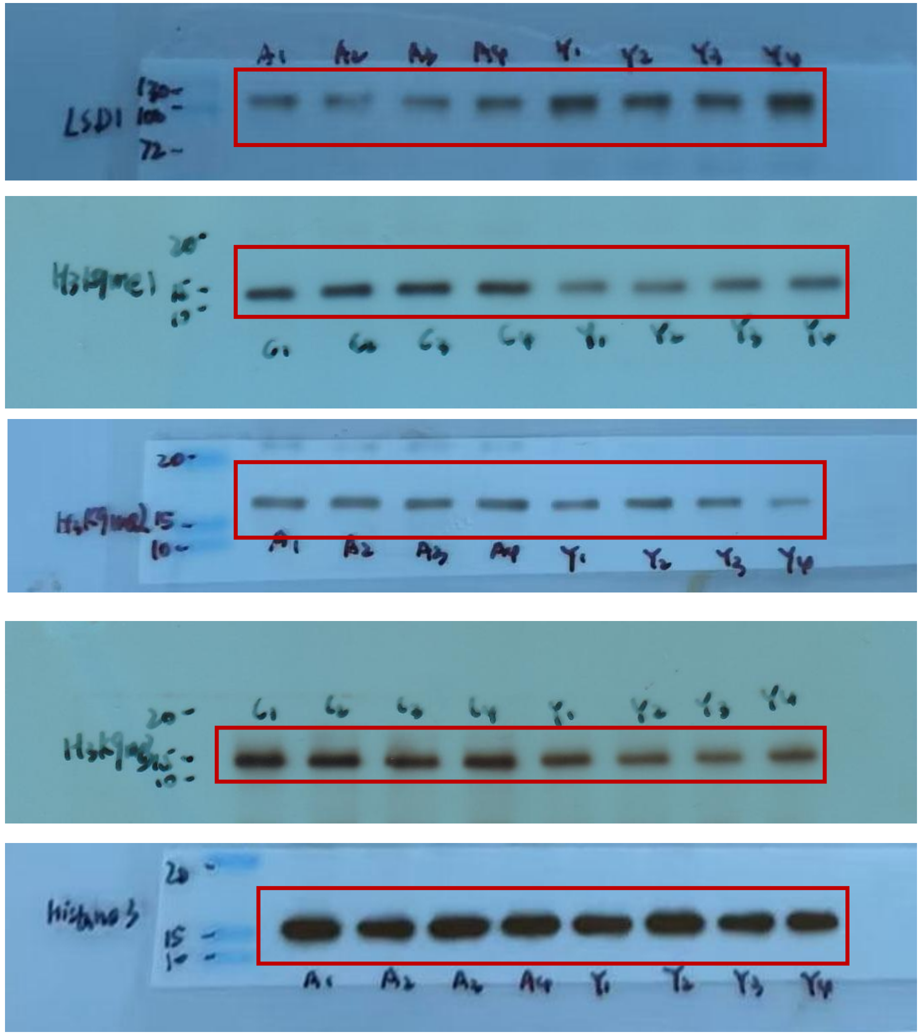

Figure 4I

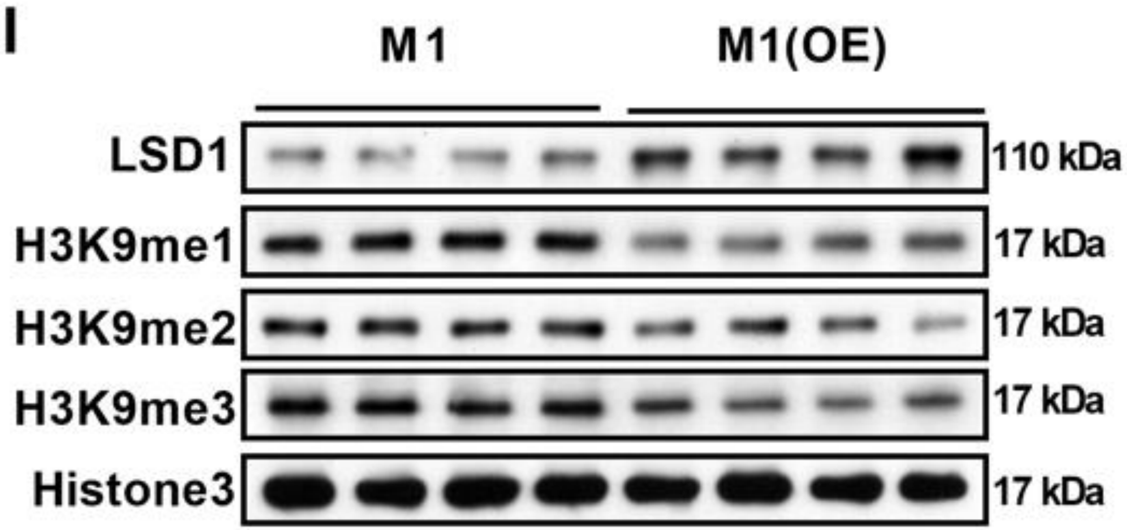
I
M1
M1(OE)
LSD1
H3K9me1
H3K9me2
H3K9me3
Histone3
110 kDa
17 kDa
17 kDa
17 kDa
17 kDa

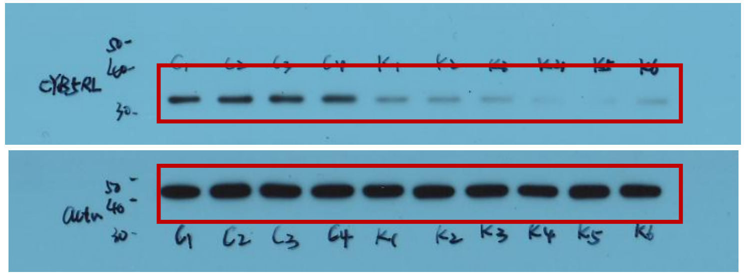

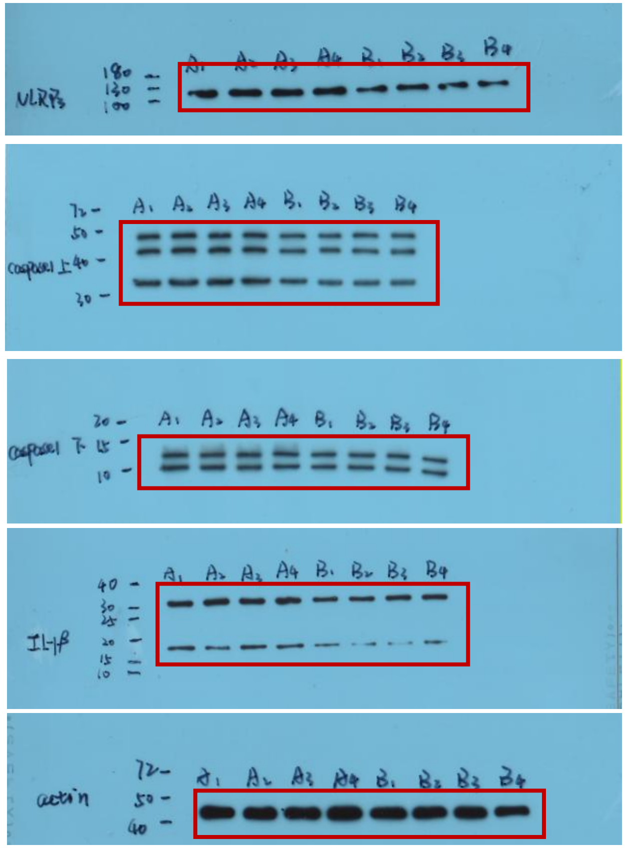

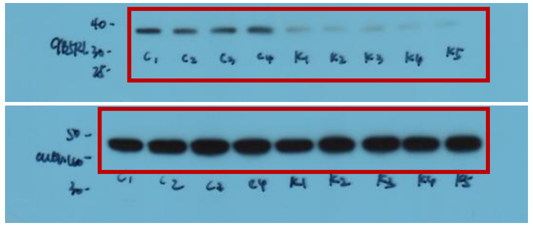

Figure 5A

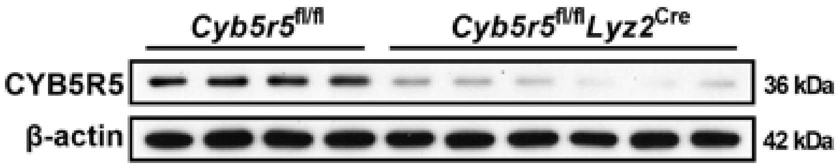

Figure 5C

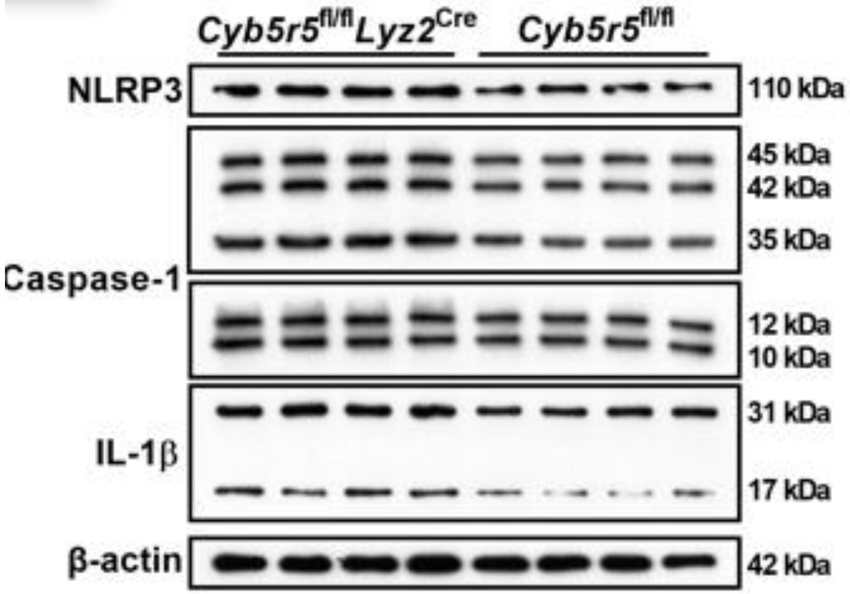

Figure 5E

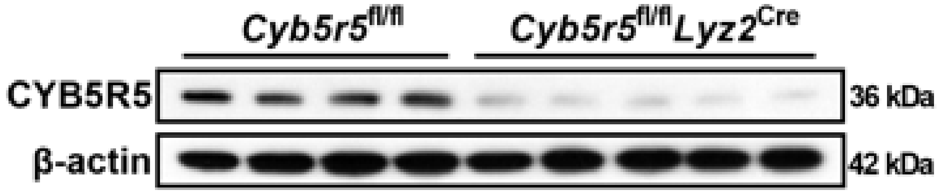

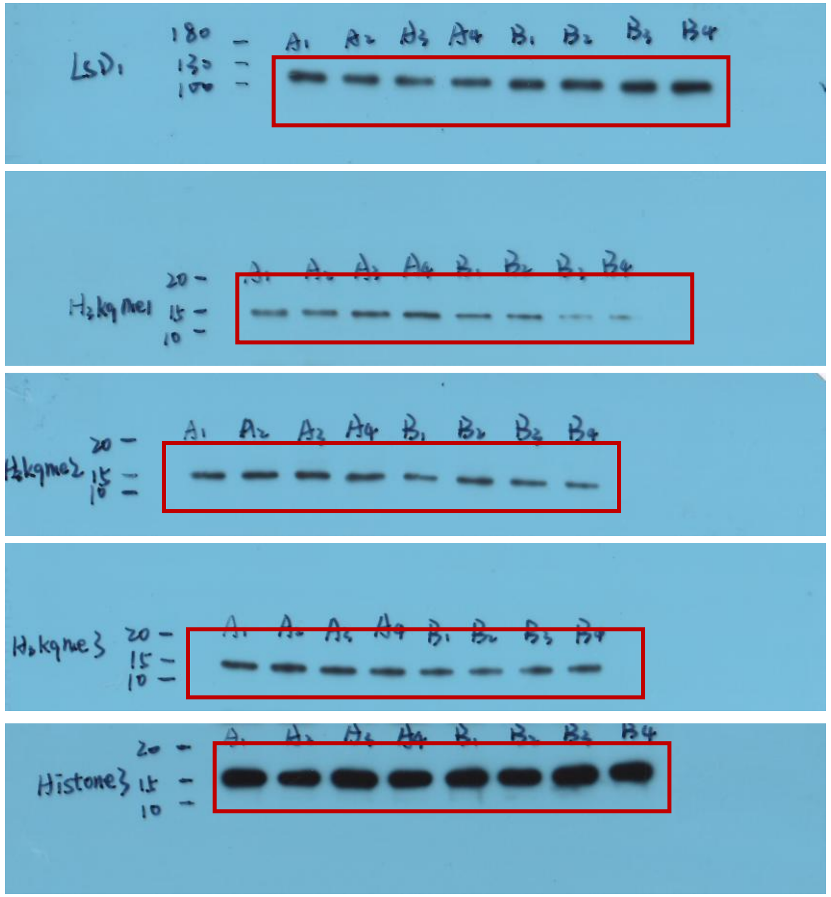

Figure 6D

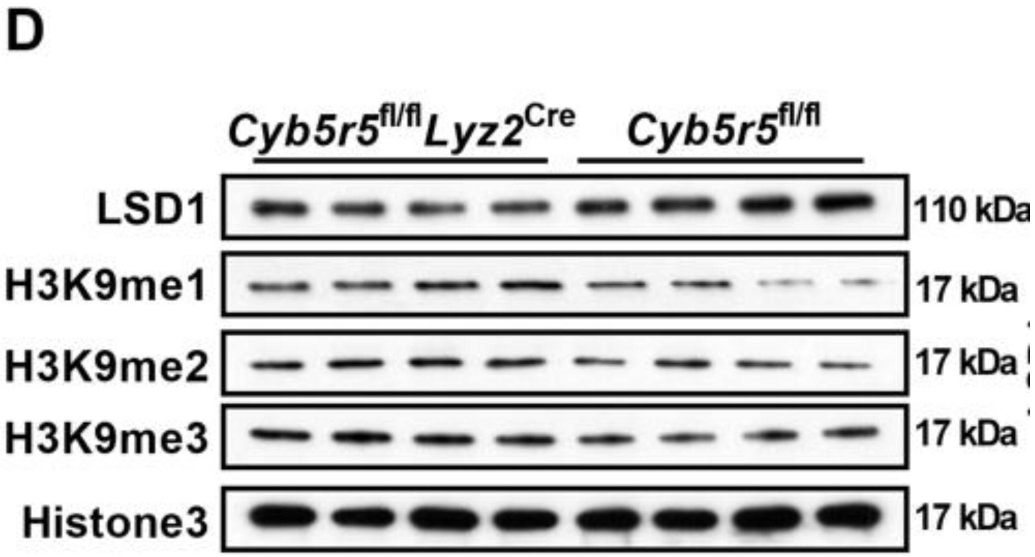

D
Cyb5r5fl/flLyz2Cre
Cyb5r5fl/fl
LSD1
H3K9me1
H3K9me2
H3K9me3
Histone3
110 kDa
17 kDa
17 kDa
17 kDa
17 kDa

Supplementary figure 1J

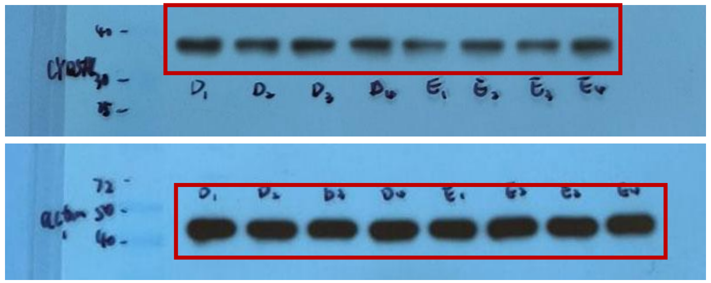

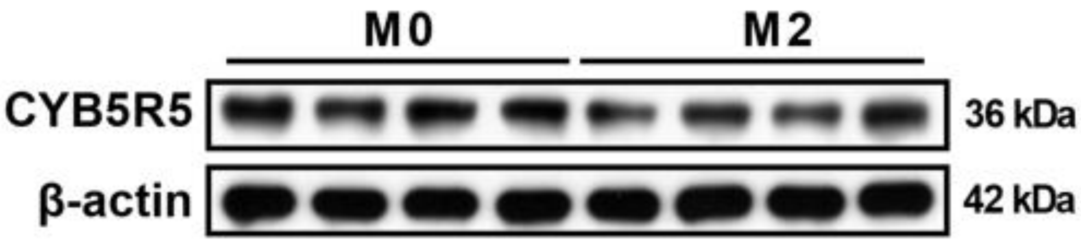

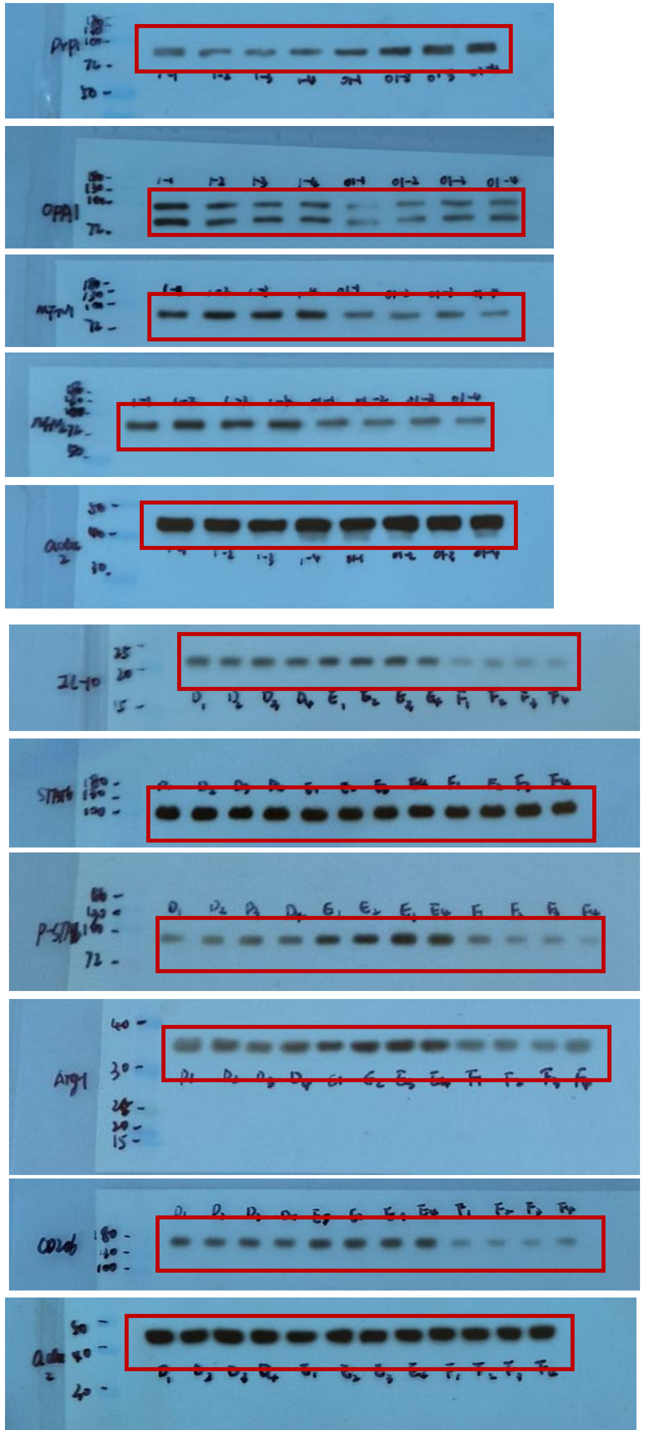

Supplementary figure 2F

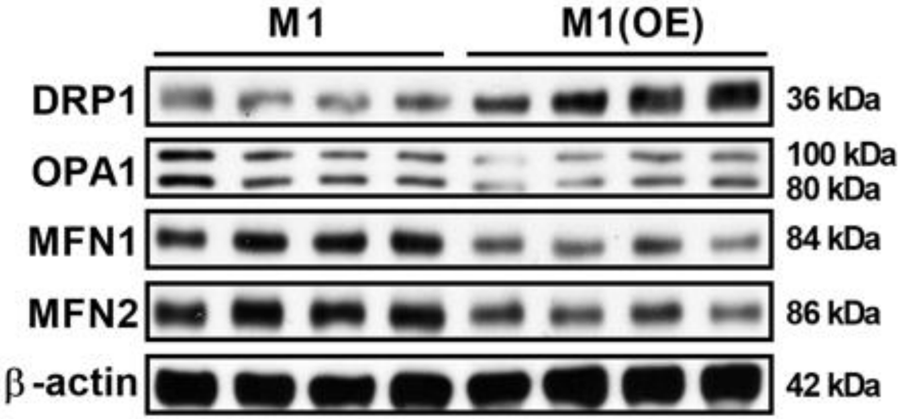

Supplementary figure 2H

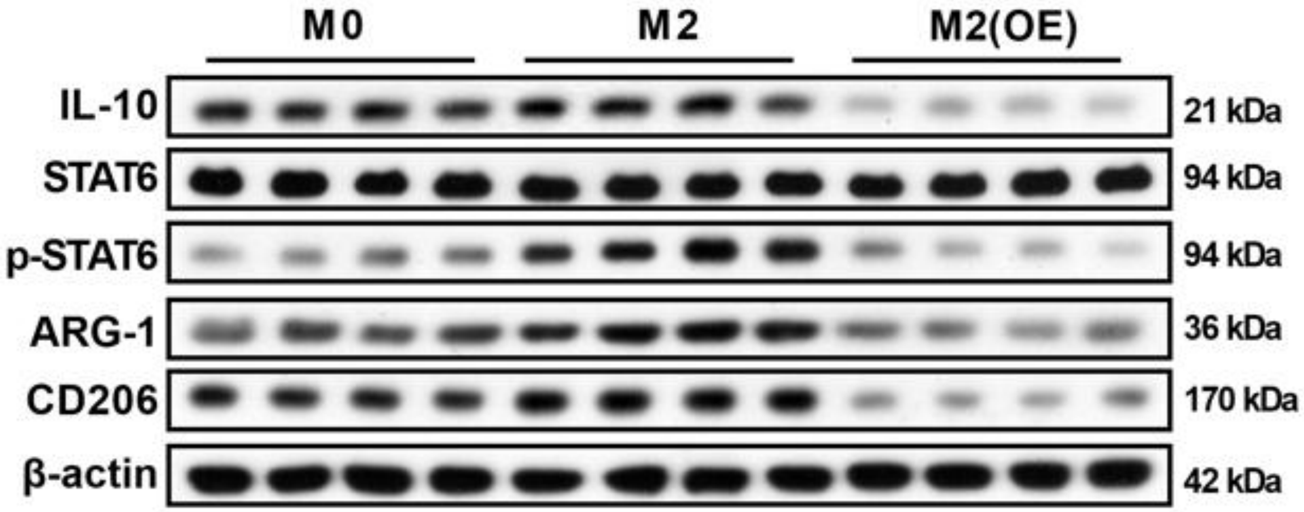

Supplement: Supplementary file 3 — Original western blots [file 41419_2025_8073_MOESM3_ESM.pdf]
